# Effects of St. John’s Wort extract on the release of paracrine markers and neurite outgrowth in mouse hippocampal HT-22 neuronal cells co-cultured with SIM-A9 microglia

**DOI:** 10.3389/fphar.2026.1916960

**Published:** 2026-10-21

**Authors:** Gabriel A. Bonaterra, Laura Much, Josephine Hahn, Heba Aziz-Kalbhenn, Olaf Kelber, Konstanze Bedal, Hans Schwarzbach, Anja Schwarz, Ralf Kinscherf

**Affiliations:** 1 Department of Medical Cell Biology, Institute for Anatomy and Cell Biology, Marburg University, Marburg, Germany; 2 Bayer Consumer Health Division, Phytomedicines Supply and Development Center, Steigerwald Arzneimittelwerk GmbH, Darmstadt, Germany

**Keywords:** antidepressant, hippocampal HT-22 neuronal cells, *Hypericum perforatum* L, neuronal plasticity, SIM-A9 Microglia, St. John’s wort

## Abstract

**Introduction:**

*Hypericum perforatum* L., commonly known as St. John’s wort (SJW), has traditionally been used to treat nervous disorders, including depression. Exposure to chronic stress events can affect neuronal and microglial function and induce depression. However, the molecular mechanisms underlying these processes are not entirely understood. Our investigation aimed to determine the effects of STW3-VI, a special SJW extract, on the paracrine signaling between microglia and neurons *in vitro*.

**Methods:**

Co-cultures of mouse SIM-A9 microglia (SIM-A9) with mouse hippocampal HT-22 neuronal cells (HT-22) were treated with different concentrations of SJW, hyperoside, an active component of SJW. Escitalopram, or nerve growth factor (NGF) as a positive control. To further elucidate the paracrine signaling, cell viability, ELISA, neurite outgrowth, migration, and ProteomeProfile™ methods were applied.

**Results:**

Incubation HT-22 with SJW, escitalopram, hyperoside, or NGF, co-cultured with SIM-A9, stimulated neurite formation compared to the negative control. Using as screening ProteomeProfiler™ and to validate ELISA analyses, in the presence of mouse SIM-A9, SJW at a concentration 0.50 μg/mL, as well as 0.019 μg/mL hyperoside or 2.10 μg/mL escitalopram, significantly increased the cholinergic differentiation factor (CDF/LIF) release compared to the respective control. After treatment of mouse hippocampal HT-22 with escitalopram, hyperoside, or NGF, the release of the anti-inflammatory cytokine IL-10 was increased. Interestingly, SJW and hyperoside inhibited the release of growth differentiation factor 15 (GDF-15) in co-culture. All experiments were performed under unstimulated co-culture conditions.

**Conclusion:**

Our data show that, under unstimulated co-culture conditions without external stimulation, an increase in CDF/LIF levels in HT-22 cells in the presence of SIM-A9 cells, a reduction in GDF-15 release by SIM-A9 microglia, and stimulation of neurite growth may indicate a possible effect of SJW on paracrine release and neurite outgrowth. Furthermore, the results indicate that hyperoside, escitalopram, and NGF enhance IL-10 release. Since our findings were derived from *in vitro* experiments, we can only speculate that a similar effect might occur in patients with depressive disorders. Additional *in vitro*, *in vivo*, and clinical studies are necessary to confirm this hypothesis.

## 1 Introduction

Major depressive disorder (MDD) is a condition that affects both mental and physical wellbeing, representing a significant public health concern for millions (Liu et al., 2020). Common clinical symptoms include persistent low mood, loss of appetite, decreased interest in activities, sleep disturbances, and, in severe cases, suicidal behavior (Pollak et al., 2010). Three main hypotheses regarding MDD have been proposed: depletion of monoamine neurotransmitters, decreased neuroplasticity, and alterations in the hypothalamic-pituitary-adrenal (HPA) axis (Jesulola et al., 2018). However, these hypotheses face challenges in adequately explaining the specific pathological mechanisms, particularly in identifying non-neuronal cells of the central nervous system (CNS) involved in MDD (Liu et al., 2017).

Chronic stress and the resulting neurological damage are key factors in the pathophysiology of depression (Lazaratou et al., 2010). In this context, microglia influence neuronal plasticity and play a crucial role in the repair processes of individual neurons throughout both early and adult life, shaping and sculpting the CNS (Ferrini and De Koninck, 2013). Microglia are an integral part of the innate immune system in the CNS, regulating synaptic microenvironment changes and synapse formation and pruning in both healthy and diseased brains (Deng et al., 2020). Disruptions in communication between microglia and neurons are linked to several brain pathologies, including depression (Yirmiya et al., 2015; Hellwig et al., 2016). Therefore, microglial dysfunction likely causes disorders in synaptic regulation, contributing to major depression (Kwon et al., 2017). However, the cellular and molecular mechanisms underlying MDD remain unclear. Considering MDD as a microglia-associated disorder, evidence suggests that microglia regulate neuronal activity and synaptic plasticity by controlling neuroinflammation (Santos et al., 2016; Troubat et al., 2021; Cornell et al., 2022). Paracrine signaling via a secreted soluble ligand is a predominant mechanism of microglia-neuron crosstalk, bidirectional and often reciprocal (Haidar et al., 2022). Microglia and neurons release soluble ligands that bind to receptors on microglia or neurons; these include neurotrophic factors as well as pro-inflammatory factors that have harmful effects on neurons (Haidar et al., 2022). This interaction includes the neuronal fractalkine (CX3CL1)/microglial fractalkine receptor (CX3CR1) proteins (Paolicelli et al., 2011), as well as microglia and glutamatergic neurons, which express brain-derived neurotrophic factor (BDNF) (Andreska et al., 2014; Parkhurst et al., 2013). BDNF is a protein that plays a crucial role in processes such as neuroplasticity, synaptic modulation, and neuronal survival (Correia et al., 2023). BDNF is involved in the pathophysiology of disorders such as MDD (Correia et al., 2023). Accumulating evidence suggests that BDNF serves as a transducer between antidepressant drugs and neuroplastic changes resulting in the improvement of depressive symptoms (Björkholm and Monteggia, 2016). Antidepressants, including selective serotonin reuptake inhibitors (SSRIs) such as escitalopram, influence microglial activation, reduce neuroinflammation, and regulate BDNF, thereby improving depressive behavior (Dionisie et al., 2021). In this context, cholinergic differentiation factor (CDF/LIF) and growth differentiation factor-15 (GDF-15) are involved in regulating inflammation and neuroprotection in the CNS (Davis and Pennypacker, 2018; Isik et al., 2024), playing a direct role in maintaining the integrity of the neuroglia for cognitive and mental health (Metcalfe et al., 2017). CDF/LIF is a critical paracrine signaling molecule crucial to the CNS and GDF-15 acts in an autocrine/paracrine manner when secreted by stressed or damaged cells, protecting and locally influencing neighboring cells (Lin et al., 2020; Rochette et al., 2021). *Hypericum perforatum* L., commonly known as St. John’s wort (SJW), has been used for centuries to treat mild to moderate depression with minimal side effects (Gastpar et al., 2006). In Germany, SJW is available as Laif®900 (Steigerwald Arzneimittelwerk GmbH, Darmstadt, Germany) in pharmacies. Previous studies involving mouse hippocampal HT-22 neuronal cells have described a protective effect of SJW against glutamate-induced cytotoxicity (Breyer et al., 2007). Furthermore, SJW has been shown to protect microglia from oxidative stress and NMDA- or glutamate-induced cytotoxicity and to possess anti-inflammatory properties (Bonaterra et al., 2020). Using *in vitro* and *in vivo* models of Alzheimer’s disease, it has been demonstrated that SJW extracts can rescue neurons, enhance memory, and activate microglia (Hofrichter et al., 2013). Previously, we showed that SJW stimulates neurite formation in differentiated hippocampal neurons and protects against glutamate or N-methyl-D-aspartate (NMDA)-induced cytotoxicity (Bonaterra et al., 2018). Moreover, regarding antioxidant defenses, SJW increased intracellular reduced glutathione levels compared with glutamate or NMDA. Additionally, SJW exhibits anti-inflammatory properties against lipopolysaccharide (LPS)-induced macrophage activation (Bonaterra et al., 2018). We have demonstrated that SJW enhances migratory abilities and increases phagocytic capacity in mouse SIM-A9 microglia (Bonaterra et al., 2020). In this context, one of the primary functions of microglia is to identify and remove unnecessary neural connections through phagocytosis, indicating that microglia actively remove synapses (Brioschi et al., 2020), thus playing a key role in neuronal plasticity through synaptic refinement and modulation of brain connectivity (Liu et al., 2021). This process promotes the development of neural circuits, thereby enhancing neuronal network efficiency (Liu et al., 2021). However, the paracrine signals that microglia and neurons use to regulate synaptic pruning and neuronal plasticity remain unknown. As others have shown, natural compounds such as hypericin appear to act as neuroprotective agents, supporting the survival of both neurons and glial cells (Farzadmanesh et al., 2025; Suswidiantoro et al., 2026). Little is known about the interactions between hippocampal neurons and microglia, and their possible relationship to depression. Our previous findings, published on SIM-A9 microglia and HT-22 neuronal cells, 2 cell types found in close contact in the CNS, indicate neurite outgrowth and activation of anti-inflammatory properties, accompanied by improved migratory capacity. An important aspect yet to be investigated is how the interaction between these 2 cell types affects these parameters, characteristics of neural plasticity. Therefore, this study aimed to investigate the effects of STW3-VI, an SJW extract, on the interaction between hippocampal HT-22 neuronal cells and SIM-A9 microglia under unstimulated co-culture conditions.

## 2 Materials and methods

### 2.1 Substances under test

Steigerwald Arzneimittelwerk GmbH (Darmstadt, Germany) provided the STW3-VI, the active component of the phytopharmaca Laif®900, a special St. John’s wort (SJW) extract of *H. perforatum* L. dry extract. *Hypericum perforatum* L was identified and named according to the Kew Medicinal Plant Names Service (Dauncey et al., 2019). The extraction and chemical characterization methods have already been extensively described previously (Breyer et al., 2007; Grundmann et al., 2010; Jungke et al., 2011; Bonaterra et al., 2020). The analysis of SJW (dry extract, lot 0,100,692,001) was conducted using High-Performance Thin-Layer Chromatography (CAMAG® HPTLC, Muttenz, Switzerland), as presented in Supplementary Figure S1. Reproducibility was confirmed by analysis of hyperoside and rutin components of lot 0100692001, compared with other lots, in Supplementary Figure S2. The total extract contains 9.9%–10% flavonoids. The 9.9%–10% flavonoid fraction contains 37% hyperoside, or 3.7% hyperoside in the total extract. The amount of hyperoside was calculated in the total extract; for example, 1 µg of extract contained 0.1 µg of flavonoids and 0.037 µg of hyperoside. The main components of SJW, namely flavonoids, hypericin, hyperoside, and hyperforin (which are used for internal standardization), are also shown in Supplementary Figure S1, according to the principles described in the chapter “reference standards” of the European Pharmacopoeia (2008) (Bonaterra et al., 2020).

The drug extract ratio (DER) was 3–6:1, and the extractant used was 80% ethanol. Escitalopram oxalate (EC, CAS No. 219861-08-2, Merck/Sigma-Aldrich Chemie GmbH, Munich, Germany) served as control, as previously described by others (Dionisie et al., 2021; Su et al., 2015). Hyperoside (Quercetin 3-O-galactoside; CAS No. 482-36-0. Merck/Sigma-Aldrich Chemie GmbH), one of the active components of the dry extract (Fan et al., 2017; Huang et al., 2021; Wang et al., 2022; Song et al., 2024) was included. Recombinant nerve growth factor (NGF) protein (ab69757; Abcam plc., Cambridge, United Kingdom) was used as a control for neurite outgrowth. The substances used in this study were dissolved in culture medium.

### 2.2 Cell culture

The brain-derived immortalized mouse microglia (SIM-A9 [ATCC® CRL-3265™]; Nagamoto-Combs et al., 2014) were cultured at 37 °C in a humidified atmosphere with 5% CO_2_ in Dulbecco’s Modified Eagle’s Medium (DMEM): F-12 Medium, a 1:1 mixture of DMEM and Ham’s F-12, supplemented with heat-inactivated 10% fetal bovine serum (FBS), 5% horse serum, and penicillin/streptomycin [(100 U/mL, 0.1 mg/mL) (Capricorn Scientific GmbH, Ebsdorfergrund, Germany)] (Bonaterra et al., 2020). The immortalized mouse hippocampal HT-22 neuronal cells (HT-22) were cultured in Dulbecco’s Modified Eagle’s Medium (Capricorn Scientific GmbH), supplemented with 10% fetal bovine serum (FBS), 100 U/mL penicillin, and 0.1 mg/mL streptomycin (Capricorn Scientific GmbH). HT-22 neuronal cells were used at passages 18-30, SIM-A9 microglia at passages 5-25. Prior to the co-culture investigations, preliminary experiments indicated that the optimal medium for both cell types is the one we used for HT-22 cells. Identical culture conditions were maintained across all monoculture and co-culture comparisons; however, for co-culture, both cell lines were cultured using the medium and conditions employed for HT-22 cells. Two controls were included in the experiments: control 1 (HT-22) and control 2 (co-cultured with SIM-A9 microglia), both in medium alone. Figure 1 illustrates the experimental setup.

**FIGURE 1:**
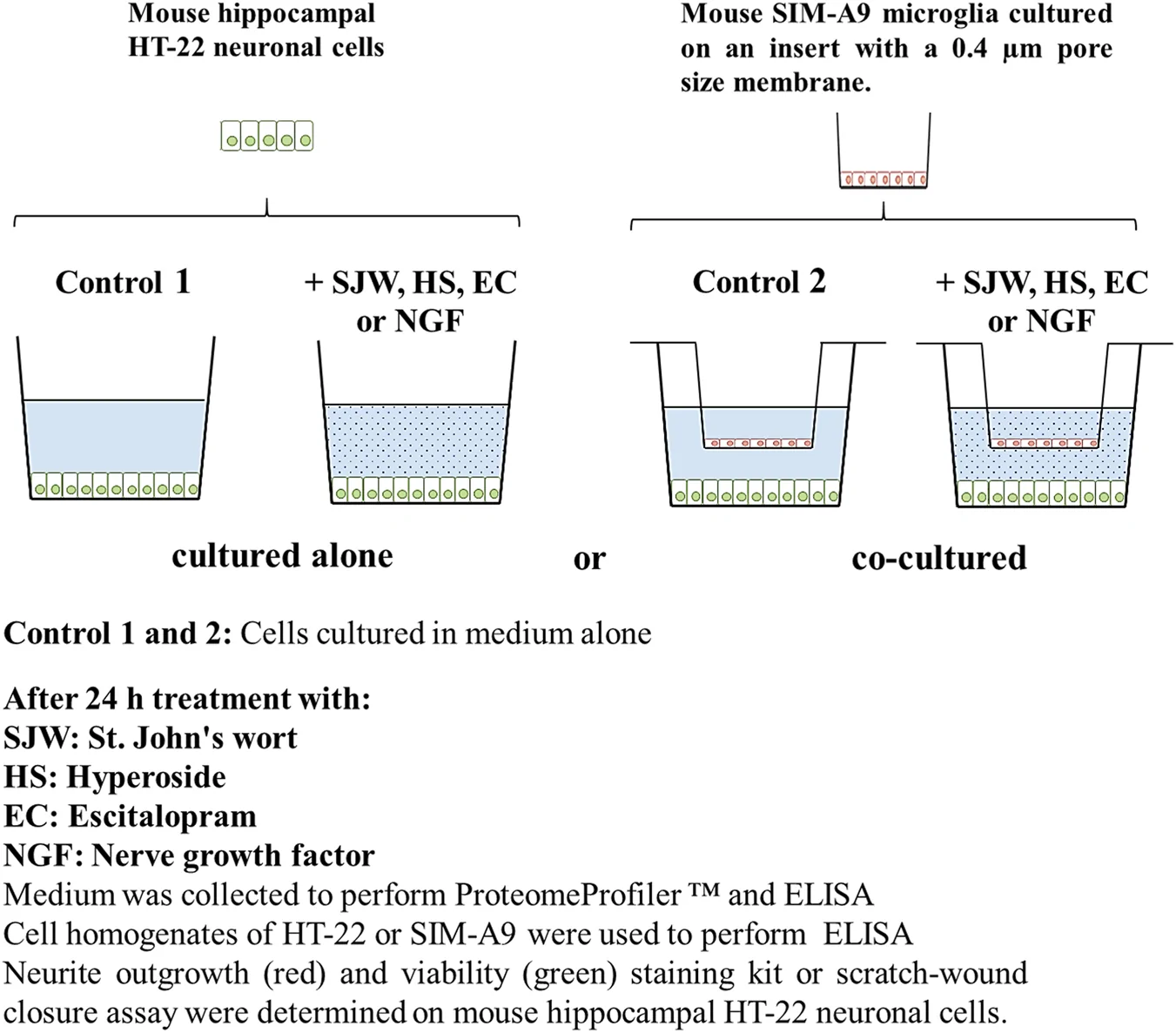
Graph of the co-culture experiments. Culture of mouse hippocampal HT-22 neuronal cells with or without inserts (containing mouse SIM-A9 microglia)**.** After 24 h, the microglia inserts were placed in the wells (containing mouse hippocampal HT-22 neurons) and treated with substances under test or cultured and treated alone (without inserts). Mouse hippocampal HT-22 neuronal cells cultured alone in medium are indicated as control 1. Mouse hippocampal HT-22 neuronal cells co-cultured with mouse SIM-A9 microglia are indicated as control 2. After 24 h of treatment, the culture medium was collected and analyzed using ProteomeProfiler® and ELISA. The cell homogenates of mouse hippocampal HT-22 neuronal cells and mouse SIM-A9 microglia were analyzed by ELISA. The living mouse hippocampal HT-22 neuronal cells were analyzed with the neurite outgrowth staining kit and scratch-wound closure assay.

To detect *Mycoplasma* sp. contamination in HT-22 and SIM-A9 cell culture samples were stained with Hoechst 33258 or DAPI (Chen, 1977) and MycoStrip® 2.0 -Mycoplasma Detection Kit (Cat.code: rep-mysv2-10; InvivoGen US, San Diego, United States). HT-22 cell-line authentication was performed using NMDA receptor immunoreactivity by immunocytochemistry and by determination of glutamate toxicity in HT-22 cells measured using PrestoBlue® Viability test (Life Technologies) (Supplementary Figure S4). The characterization of SIM-A9 was performed using a green fluorescent microspheres phagocytosis test and the response to LPS stimulation (Nagamoto-Combs et al., 2014) (Supplementary Figure S5). The morphological characteristics of HT-22 and SIM-A9 are shown in Supplementary Figures S4, S5.

### 2.3 Viability assay

Mouse SIM-A9 cells (3.0 × 10^4^ cells/well) were seeded into 96-well plates (Sarstedt AG and Co, Nürnbrecht, Germany) and 1.0 × 10^4^ cells/well mouse hippocampal HT-22 neuronal cells were plated into collagen-I-coated 96-well plates (Greiner Bio-One GmbH, Frickenhausen, Germany). After 24 h, the medium was changed, and the cells were treated with test substances for an additional 24 h. Afterward, the viability was determined using PrestoBlue™ Cell Viability Reagent (Fisher Scientific GmbH, Schwerte, Germany) according to the manufacturer’s protocol. Briefly, a final concentration of 10% PrestoBlue™ was used. After 30–60 min, the optical density (OD) was measured at 570 nm and 600 nm (as a reference) using a Sunrise ELISA reader (Tecan Group Ltd., Männedorf, Switzerland). Results were expressed as % viability = [(OD570 nm/600 nm of samples) * 100%]/OD570 nm/600 nm of control 1 (Figure 1). Additionally, when necessary, cells were fixed with 4% PFA-PBS and afterward stained with crystal violet solution (0.04% crystal violet in 4% [v/v]) (Merck KGaA, Darmstadt, Germany), solubilized with 1% SDS/PBS, and the absorbance was measured at 595 nm and 660 nm (as a reference) (Figure 1).

### 2.4 Co-culture of mouse hippocampal HT-22 neuronal cells and mouse SIM-A9 microglia

Mouse hippocampal HT-22 neuronal cells (2.9 × 10^3^ cells/well) were seeded in 24-well plates coated with Bio-One CELLCOAT™, which was coated with rat tail Type I collagen (Greiner Bio-One GmbH, Frickenhausen, Germany). Mouse SIM-A9 cells (3.0 × 10^3^ cells/insert) were used in a 24-well format with a 0.4 µm pore size PET track-etched membrane (Corning Inc., NY, United States). After 24 h, mouse SIM-A9 microglia inserts were placed into wells containing mouse hippocampal HT-22 neuronal cells. After another 24 h, they were co-cultured and treated with the substances under investigation. Based on viability assays, non-cytotoxic concentrations were used in the co-culture experiments. Briefly, treatment was performed with or without SJW, escitalopram oxalate (Merck/Sigma-Aldrich Chemie GmbH); hyperoside (HS, Merck/Sigma-Aldrich Chemie GmbH); or NGF (Abcam plc). The culture medium was collected for analysis using the Mouse XL Cytokine Array Kit Proteome Profiler™ Array and ELISA Duo Set (R&D Systems Europe, Ltd., Abingdon, United Kingdom) (Figure 1).

### 2.5 Determining the neurite outgrowth process

Neurite outgrowth in HT-22 was studied using the Neurite Outgrowth Staining Kit (catalog no. A15001 Thermo Fisher Scientific Inc., Rockford, United States), according to the manufacturer’s guidelines. This kit includes two dyes: One for staining the cell membrane and another for indicating cell viability, both of which can be measured simultaneously.

In summary, the dyes were diluted in Hank’s solution (Capricorn Scientific GmbH, Ebsdorfergrund, Germany), and 200 μL was gently added to each well, followed by incubation at 37 °C for 15 min. After this, a background suppression dye (diluted in Hank’s solution) was added. Images were taken using an Axiovert 135 microscope equipped with an AxioCam MRm Camera (Carl Zeiss, AG) and a fluorescence filter. The specific cell membrane dye was excited at 555 nm with an emission peak of 565 nm, while the viability indicator was excited at 495 nm with an emission peak of 515 nm. Representative images were obtained using a fluorescence microscope (Axiovert 135; Carl Zeiss AG, Oberkochen, Germany), but they were not used to quantify neurite outgrowth. Quantification of relative fluorescence was performed using a monochromator fluorescence plate reader, Cytation™ 3 Cell Imaging Multi-Mode Reader (BioTek Instruments, Inc., Winooski, United States), at 554 nm excitation and 567 nm emission (cell membrane stain) and 483 nm excitation and 525 nm emission (cell viability indicator), (Hancock et al., 2015; Broeglin et al., 2026) (Figure 1).

### 2.6 Determination of the migratory capacity

A scratch-wound closure assay was performed to examine the effects of SJW on the migratory ability of HT-22 in co-culture with SIM-A9 (Figure 1). Culture silicon inserts (2-well) for self-insertion (Ibidi GmbH, Gräfelfing, Germany) were used to make the scratch. Mouse HT-22 (2.9 × 10^3^ cells/well) were seeded in a 24-well plate and allowed to grow for 24 h. The silicon inserts were then removed, and the scratches were treated with SJW, hyperoside, escitalopram, or NGF. The inserts were cultured with or without mouse SIM-A9 (3.0 × 10^3^ cells/insert) (Figure 1). The wells were photographed immediately (at 0 h) and after 3, 6, 10, and 12 h of treatment using an inverted Axiovert 135 microscope with a motorized stage and a digital AxioCam MRc camera (Carl Zeiss AG, Oberkochen, Germany). The areas of the scratches were measured using ImageJ/FIJI freehand selection and area measurement. The migration of HT-22 into the scratch (wound closure) was calculated as a percentage of the area at the 0-h time point: % wound closure = {[(scratch area at 0 ℎ) – (scratch area after x h)] × 100%}/scratch area at 0 ℎ. One researcher performed quantification, and some of the images were randomly selected and analyzed by another researcher.

### 2.7 Enzyme-linked immunosorbent assay (ELISA)

Cytokine release into the culture medium was measured by ELISA. After treating HT-22 with the test substances, with or without SIM-A9 microglia, the culture medium was harvested and frozen at −20 °C. Additionally, after treating HT-22 and SIM-A9, the cells were lysed using radioimmunoprecipitation assay (RIPA) buffer (pH 7.5; Cell Signaling Technology, Frankfurt, Germany) containing protease/phosphatase inhibitors (Cell Signaling Technology).

Protein concentrations were determined spectrophotometrically using the bicinchoninic acid (BCA) protein assay (Thermo Scientific, Rockford, United States) and used to normalize the cytokine concentrations determined by ELISA. The cell lysate was frozen at −20 °C. According to the manufacturer’s instructions, cytokines in the culture medium and the cell lysate were quantified using the DuoSet ELISA Development kit (R&D Systems Europe, Ltd.). NUNC MaxiSorp™ microplates (Thermo Fisher Scientific), containing 96 wells, were utilized. First, the appropriate dilution factor was determined to ensure the samples were within the assay linear range. Each sample was analyzed with two replicates per treatment. The reaction was visualized using the peroxidase substrate Sigma Fast™ o-phenylenediamine dihydrochloride (OPD, Merck/Sigma-Aldrich Chemie GmbH) for 30–45 min and then stopped with 25 μL of 3 N HCl. Absorbance was measured at 490 nm with a reference at 655 nm. The cells were fixed with 4% PFA-PBS and afterward stained with crystal violet solution (0.04% crystal violet in 4% [v/v]) (Merck KGaA), solubilized with 1% SDS/PBS, and the absorbance was measured at 595 nm and 660 nm (as a reference) (Figure 1). The ELISA absorbance was normalized against crystal violet. The amount of released cytokines was determined from a standard curve, which was then used to calculate concentrations by linear interpolation. DuoSet® ELISA was used to quantify: human/mouse BDNF (cat. no. DY248; assay range 23.4–1500 pg/mL), mouse growth/differentiation factor 15 (GDF-15, cat. no. DY6385; assay range 7.81–500 pg/mL), mouse interleukin 6 (IL-6, cat. no. DY406; assay range 15.6–1000 pg/mL), mouse interleukin 10 (IL-10, cat. no. DY417; assay range 31.2–2000 pg/mL), mouse leukemia inhibitory factor (LIF, cat. no. DY449; assay range 6.25–400 pg/mL) also cholinergic differentiation factor (CDF), mouse tumor necrosis factor-alpha (TNF-α, DY410; assay range 31.2–2000 pg/mL).

### 2.8 Determination with proteome Profiler™

The harvested medium from three experiments (Figure 1) was collected, pooled, and used for the semiquantitative determination of mouse cytokines and chemokines using a commercially available Mouse XL Cytokine Array Kit Proteome Profiler™ Array (R&D Systems Europe, Ltd.) according to the manufacturer’s instructions (Abdel-Aziz et al., 2016). Briefly, treatment medium from three independent experiments was pooled and incubated on Proteome Profiler™ nitrocellulose membranes containing the 111 captured antibodies in duplicate spots for 2 h with slow stirring on a rocking platform shaker, followed by 14 h without stirring in a wet chamber. The two steps were performed in a cold room at 4 °C. The membranes were incubated with a detection antibody diluted in the assay buffer for 1 h at room temperature (RT), then with streptavidin-horseradish peroxidase (HRP) in the same buffer for 30 min at RT. Immunoreactive spots on the nitrocellulose membranes were detected using the horseradish peroxidase (HRP)-chemiluminescent substrate reagent mix (Kit Proteome Profiler™: chemi reagent 1 + 2) and then documented using the Fusion-SL Advance™ imaging system (PEQLAB GmbH, Erlangen, Germany). Images were taken at multiple exposure times. Regions of interest (ROI) were drawn around each analyte, and pixel densities from positive spots were quantified using ImageJ/FIJI software from the National Institutes of Health (Bethesda, United States). Dot-pixel intensity values were averaged, and the background value was subtracted from each dot. Representative photos of the Proteome Profiler™ membranes are presented in Supplementary Figure S3. Markers showing a difference of more than 25% in pixel intensity (after background subtraction) between control and treatment groups-and bearing a hypothetical relationship to depressive processes-were considered candidates for confirmation via ELISA (Supplementary Figure S6). This selection criterion was previously established according to the manufacturer’s recommendations. The exceptions were the cytokines TNF-α and IL-6, which served as indicators that the experiments were conducted under unstimulated co-culture conditions. Quantitative ELISA was subsequently used to validate the ProteomeProfiler™ screening results.

### 2.9 Statistical methods

SigmaPlot® 12.0 (Systat Software Inc., Chicago, United States) was used for statistical analysis. Statistical significance was determinated using one-way analysis of variance (ANOVA) with corresponding *post hoc* tests. First, we tested the data for normality using the Kolmogorov-Smirnov’s test and for equal variance using Levene’s test. Dunn’s or Dunnett’s *post hoc* tests was applied. If the data failed the normality and/or equal-variance tests, the Kruskal-Wallis test was used. Results are presented as means ± standard error of the mean (SEM). We considered results statistically significant when *p* ≤ 0.05.

## 3 Results

### 3.1 Effects of SJW, hyperoside, and escitalopram on the viability of mouse hippocampal HT-22 neuronal cells and mouse SIM-A9 microglia

First, we investigated the effects of different concentrations of SJW extract (0.25, 0.50, 1.00, 2.00, 5.00, 10.00, and 20.00 μg/mL), the correspondenting concentrations of hyperoside contained in the extract (0.0094, 0.019, 0.038, 0.094, 0.19, 0.38 and 0.75 μg/mL), and escitalopram at 5, 10, 20, 40, 80 and 100 µM (corresponding concentrations in µg/mL, 2.1, 4.15, 8.29, 16.58, 33.15 and 41.44). Escitalopram was used as an antidepressant control of mouse hippocampal HT-22 neuronal cells. SJW (5.0, 10.0 and 20 μg/mL) significantly (*p* ≤ 0.01) reduced viability by 13.2% (2.0 μg/mL), 11.2% (5.0 μg/mL), and 9.9% (10.0 μg/mL) in HT-22 compared to the medium control, which represents 100% viability (Figure 2A). Treatment of mouse HT-22 with hyperoside significantly (*p* ≤ 0.011) inhibited viability by 9.7% (0.094 μg/mL), 12.7% (0.19 μg/mL), and 9.6% (0.38 μg/mL) compared to the control (Figure 2B). In contrast, treatment with escitalopram was not cytotoxic to mouse hippocampal HT-22 neuronal cells at the concentrations used in our experiments (Figure 2C).

**FIGURE 2:**
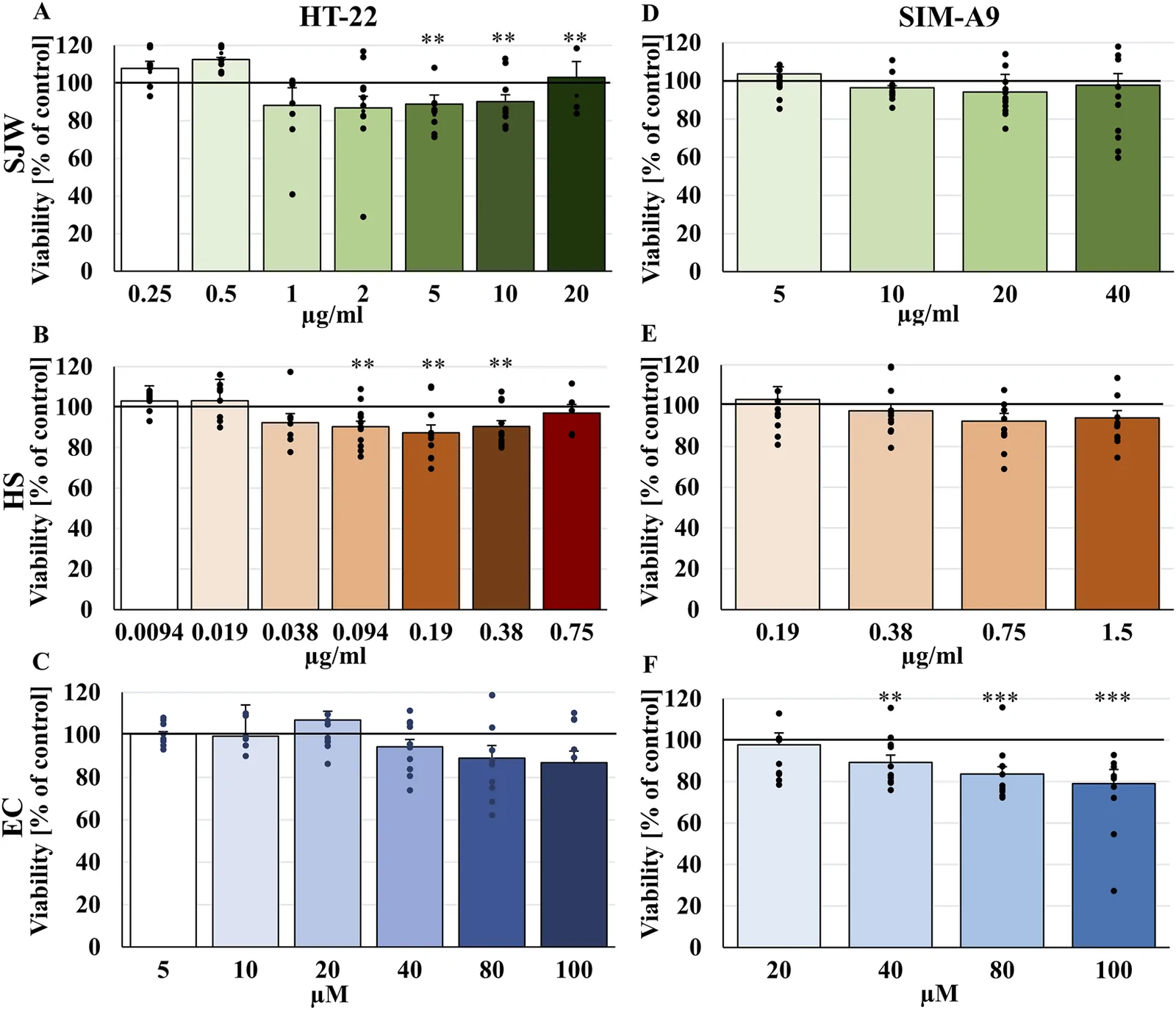
Effects of SJW, hyperoside (HS), or escitalopram (EC) treatment (24 h) on the viability of **(A–C)** mouse hippocampal HT-22 neuronal cells and **(D–F)** mouse SIM-A9 microglia. Viability was measured by PrestoBlue® assay. Values [in % viability of cells without treatment cultured in medium alone (control = 100% viability)] are given as mean +SEM; Kruskal-Wallis (*post hoc* Dunn’s method): *p*, significance, *p ≤ 0.05, **p ≤ 0.01, ***p ≤ 0.001 vs. control (100%); *n* = 5–10 independent experiments with 3 replicates per treatment.

Second, we also investigated the effects of different concentrations of SJW (5.0, 10.0, 20.0, and 40.0 μg/mL), the corresponding concentrations of hyperoside contained in the extract (0.19, 0.38, 0.75 and 1.5 μg/mL), as well as escitalopram at 20, 40, 80 and 100 µM (corresponding concentrations in µg/mL, 8.29, 16.58, 33.15 and 41.44), on the viability of mouse SIM-A9 microglia cells. We found that only escitalopram at 40, 80, and 100 µM inhibited the viability of mouse SIM-A9 microglia by 10.8% (*p* ≤ 0.01), 16.4% (*p* ≤ 0.001), and 21.0% (*p* ≤ 0.001), respectively, compared to the medium control (Figures 2D–F).

### 3.2 Effects of SJW, hyperoside, and escitalopram on neurite outgrowth of mouse hippocampal HT-22 neuronal cells

Mouse HT-22 cultured alone and treated with the test substances did not show a significantly increased neurite outgrowth compared to control 1 (Figure 3A). However, significant differences in neurite outgrowth were observed only when HT-22 were co-cultured with SIM-A9 microglia (Figure 3A). Interestingly, HT-22 co-cultured with SIM-A9 in medium (control 2) showed a 2.3-fold (*p ≤* 0.001) decrease in neurite outgrowth compared to control 1 (Figure 3A). Co-culture treated with 0.25 or 0.50 μg/mL SJW showed increased neurite outgrowth in HT-22 by 1.75-fold (*p* ≤ 0.002) and 2.10-fold (*p* ≤ 0.007), respectively, compared to control 2 (Figure 3A). Hyperoside, 0.0094 μg/mL or 0.019 μg/mL, significantly increased 2.1-fold (*p ≤* 0.002) and 1.8-fold (*p ≤* 0.027) neurite outgrowth compared to control 2 (Figure 3A). Similarly, escitalopram, at 2.10 μg/mL (5 µM) or 4.2 μg/mL (10 µM), a selective serotonin reuptake inhibitor, increased neurite outgrowth in HT-22 by 3.53-fold (*p ≤* 0.001) and 5.9-fold (*p ≤* 0.001), respectively, compared to control 2 (Figure 3A). NGF was used as a neurite outgrowth-positive control and showed significant increases of 3.22-fold and 5.14-fold in neurite outgrowth compared to control 2 (*p ≤* 0.001; Figure 3A). Figure 3B shows representative images of the neurite staining and cell viability in HT-22.

**FIGURE 3:**
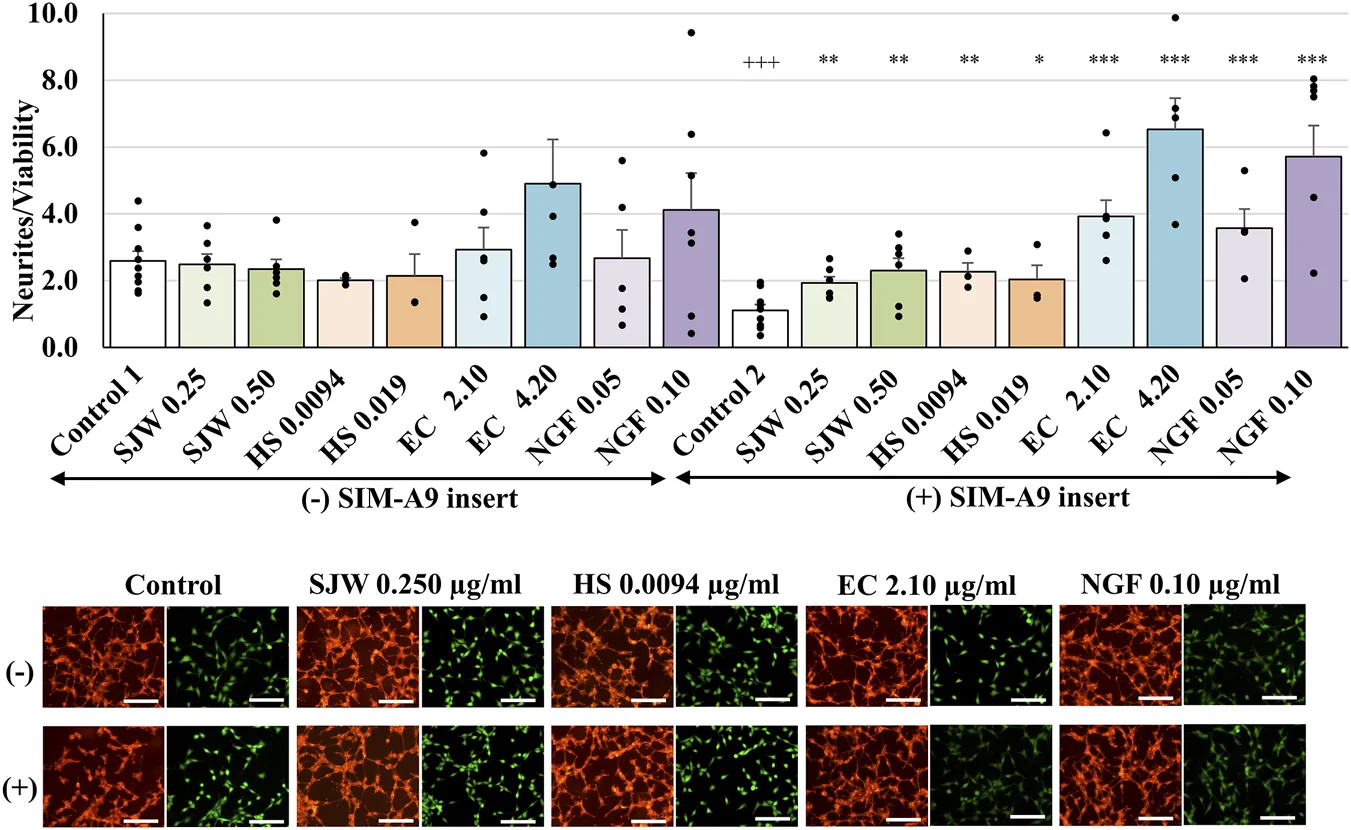
Neurite formation of mouse hippocampal HT-22 neuronal cells (with/without mouse SIM-A9 microglia) after 24 h treatment with SJW, HS, EC, or NGF. Neurite formation was **(A)** quantified, and substances under test were expressed in µg/mL, and **(B)** visualized using a neurite outgrowth (red) and viability (green) staining kit. For normalization, neurite formation values were divided by cell viability values. Data are given as mean +SEM; Kruskal-Wallis (*post hoc* Dunn’s method): *p ≤ 0.05, **p ≤ 0.01, ***p ≤ 0.001 vs. control 2 and ^+++^ *p ≤* 0.001 vs. control 1. The tested substances are given in µg/mL; *n* = 4–9 independent experiments with 2 replicates per treatment. Scale bar = 500 µm.

### 3.3 Effects of SJW, hyperoside, and escitalopram on TNF-α and IL-6 release

Microglial activation causes morphological changes, resulting in an amoeboid shape, and promotes migration to sites of inflammation (Cornell et al., 2022). They then secrete cytokines, chemokines, and reactive oxygen species (ROS), which can influence synaptic plasticity (Cornell et al., 2022). We perform the co-culture experiments under unstimulated conditions to prevent these effects. As shown by the ProteomeProfiler™ array, no change in TNF-α release was observed after treating HT-22 with the substances under test, with or without SIM-A9 (Figure 4A). To confirm these results, TNF-α release was measured using ELISA. Control 2 showed a significant increase in TNF-α levels compared to control 1 (*p* ≤ 0.001) to 25 pg/mL. This level represents the basal TNF-α release from unstimulated SIM-A9 microglia, as has already been shown by others (Nagamoto-Combs et al., 2014). Treatment with SJW, hyperoside, escitalopram, or NGF had no significant effect on the TNF-α release (Figure 4B).

**FIGURE 4:**
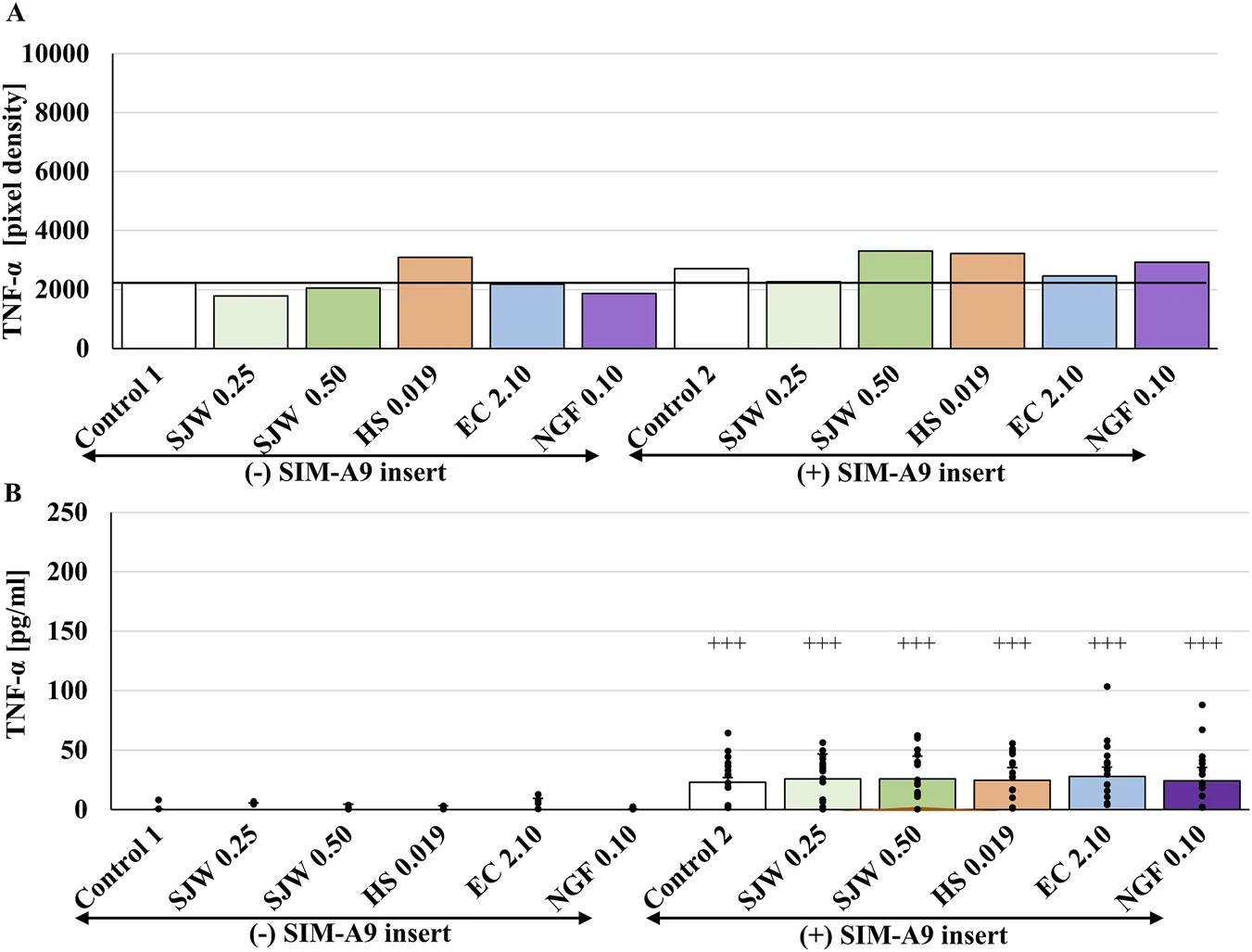
TNF-α release of mouse hippocampal HT-22 neuronal cells (with/without mouse SIM-A9 microglia) after 24 h treatment with SJW, HS, EC, or NGF determined in culture medium by **(A)** ProteomeProfiler™ a pool of samples from three experiments and **(B)** ELISA, *n* = 9 independent experiments with 2 replicates per treatment. Concentrations of TNF-α (pg/mL) were normalized with the total cell number measured by crystal violet (CV). ELISA data are given as mean +SEM; Kruskal-Wallis (*post hoc* Dunn’s method): ^+++^ *p ≤* 0.001 vs. the respective treatment in mouse hippocampal HT-22 neuronal cells cultured alone. The substances under test are given in µg/mL.

Another pro-inflammatory cytokine, IL-6, was measured using the ProteomeProfiler™ array. No change in IL-6 levels was observed after treating HT-22 with the substances under test, regardless of whether they were co-cultured with or without SIM-A9 (Figure 5A). To verify these results, IL-6 release was also determined using ELISA. Control 2 showed a significant increase in IL-6 levels compared to control 1 (*p* ≤ 0.001) to 92.6 pg/mL. This level represents the basal IL-6 release from unstimulated SIM-A9 microglia, as has recently been shown by others (Blank et al., 2022). Treatment with SJW, hyperoside, escitalopram, or NGF had no significant effect on the IL-6 release (Figure 5B).

**FIGURE 5:**
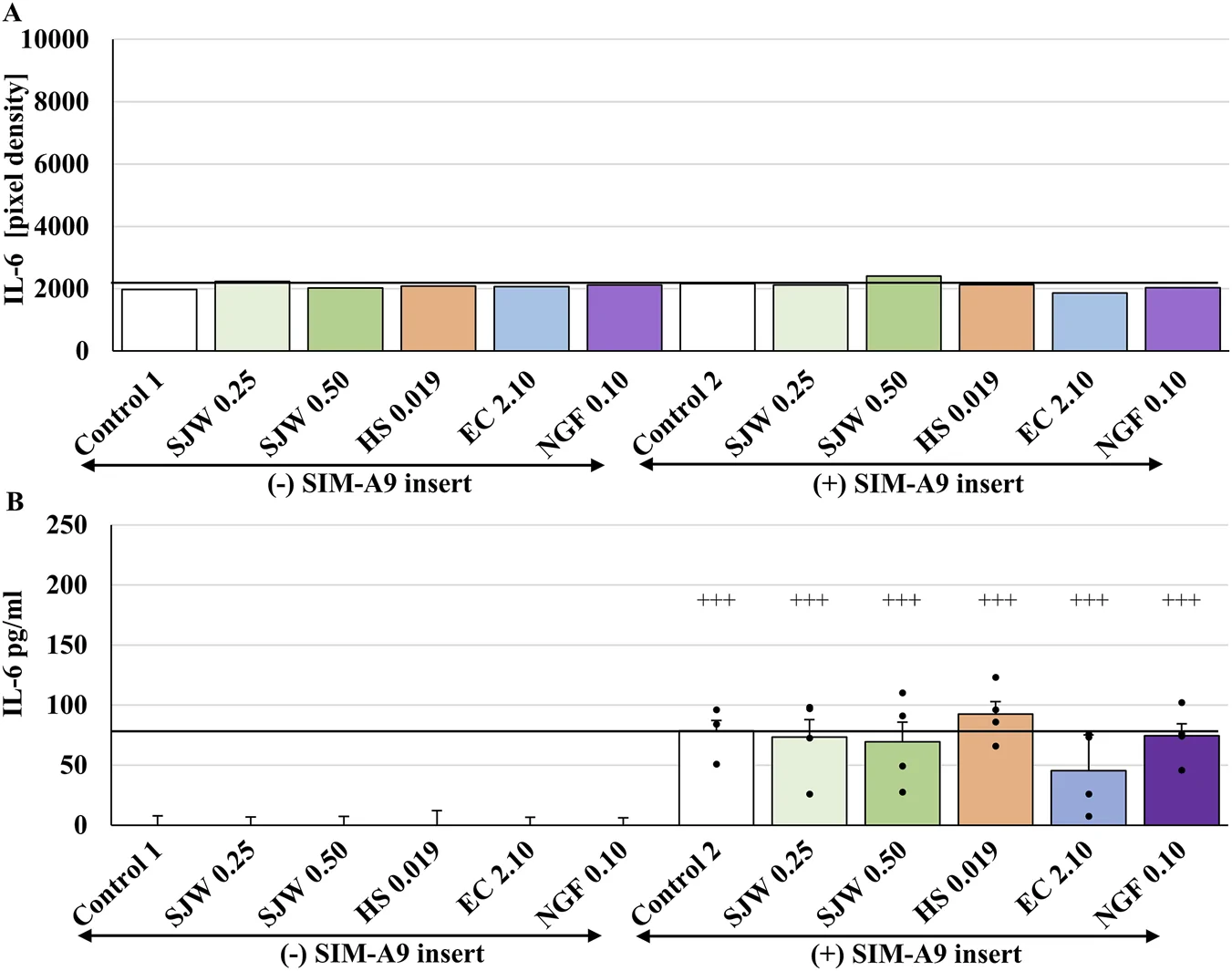
IL-6 release of mouse hippocampal HT-22 neuronal cells (with or without mouse SIM-A9 microglia) after 24 h treatment with SJW, HS, EC, or NGF, determined in culture medium by **(A)** ProteomeProfiler™ a pool of samples from three experiments and **(B)** ELISA, *n* = 4 independent experiments with 2 replicates per treatment. Concentrations of IL-6 (pg/mL) were normalized to the total cell number measured by crystal violet (CV). ELISA data are given as mean +SEM; one way ANOVA (*post hoc* Dunnett’s method): ^+++^ *p* ≤ 0.001 vs. the respective treatment in mouse hippocampal HT-22 neuronal cells cultured alone. The substances under test are given in µg/mL.

### 3.4 Effects of SJW, hyperoside, and escitalopram on BDNF release

Stress and consecutive depression reduce BDNF levels, while antidepressant treatment reverses this by raising BDNF levels (Chakrapani et al., 2020). To determine whether BDNF levels are associated with the observed variations, we measured BDNF in co-culture experiments. Treatment of HT-22 with 0.019 μg/mL hyperoside or 0.10 μg/mL NGF significantly inhibited 2.6-fold (*p* ≤ 0.027) but 0.10 μg/mL NGF 1.7-fold not significant (*p* = 0.147) the BDNF release compared to control 1 (Figure 6). The BDNF release from HT-22 co-cultured with SIM-A9 in medium (control 2) was lower by 3.5-fold (*p ≤* 0.001) than that from control 1 (Figure 6). In the co-culture of HT-22 with SIM-A9, only 2.10 μg/mL escitalopram significantly inhibited (*p* ≤ 0.044) BDNF release by 1.8-fold compared to control 2 (Figure 6). Incubation of HT-22 co-cultured with SIM-A9 with 0.25 μg/mL or 0.50 μg/mL SJW significantly inhibited BDNF release by 2.6-fold (*p ≤* 0.007) or 2.5-fold (*p ≤* 0.038) compared to the respective treatment of mouse hippocampal HT-22 neuronal cells cultured alone (Figure 6). Hyperoside did not affect BDNF release in co-culture with SIM-A9 (Figure 6). Incubation of HT-22 co-cultured with mouse SIM-A9 microglia with escitalopram (2.10 μg/mL and 4.20 μg/mL) or NGF (0.10 μg/mL) inhibited the BDNF by 3.4-fold (*p ≤* 0.007), 3.5-fold (*p ≤* 0.001), and 3.4-fold (*p ≤* 0.004) significantly, compared to the respective treatment of HT-22 cultured alone (Figure 6).

**FIGURE 6:**
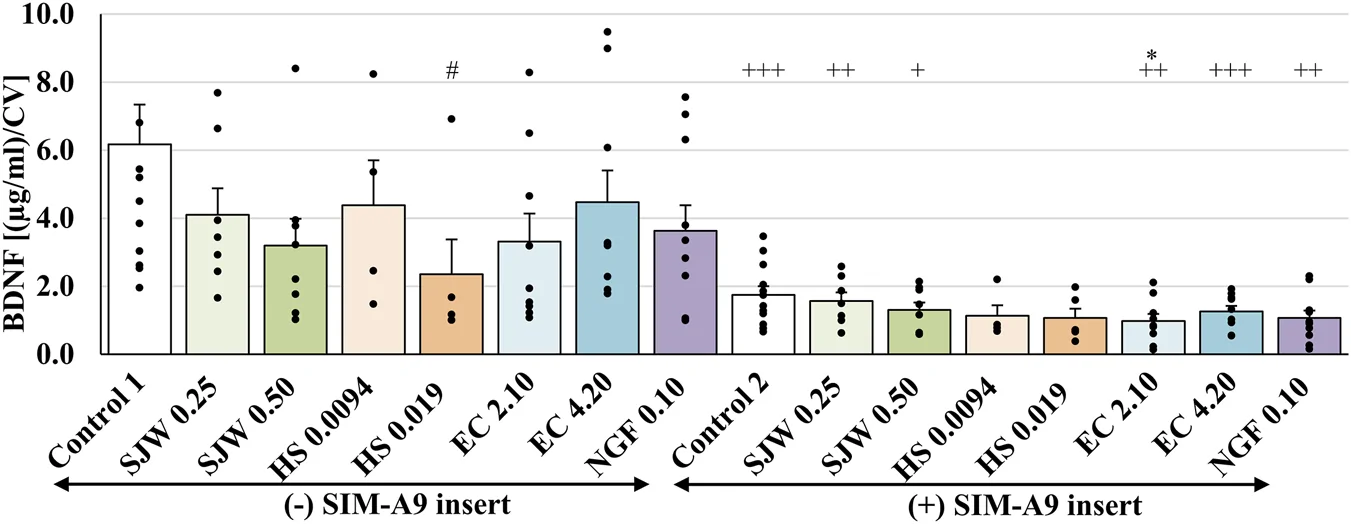
BDNF release of mouse hippocampal HT-22 neuronal cells (with or without mouse SIM-A9 microglia) after 24 h treatment with SJW, HS, EC, or NGF. Concentrations (µg/mL) of BDNF were normalized to the total cell number measured by crystal violet (CV). Data are given as mean +SEM; Kruskal-Wallis (*post hoc* Dunn’s method): ^#^ *p ≤* 0.05 vs. control 1, *p ≤ 0.05 vs. control 2, and ^+^ *p ≤* 0.05, ^++^ *p ≤* 0.01, ^+++^ *p ≤* 0.001 vs. respective treatment of mouse hippocampal HT-22 neuronal cells cultured alone. The substances under test are given in µg/mL; *n* = 5 independent experiments with 2 replicates per treatment.

### 3.5 Effects of SJW, hyperoside, and escitalopram on the migration of mouse hippocampal HT-22 neuronal cells co-cultured with mouse SIM-A9 microglia or cultured alone

Neuronal plasticity encompasses several mechanisms, including migration, proliferation, neurite outgrowth, synaptogenesis, and the maturation of functional synapses (Wainwright and Galea, 2013). To investigate the migration of mouse hippocampal HT-22 neuronal cells, we performed scratch assays in culture alone and in co-culture. Treatment of HT-22 neuronal cells with the substances under test, with or without inserts containing mouse SIM-A9 microglia, did not significantly affect their migratory capacity (Figures 7A,B).

**FIGURE 7:**
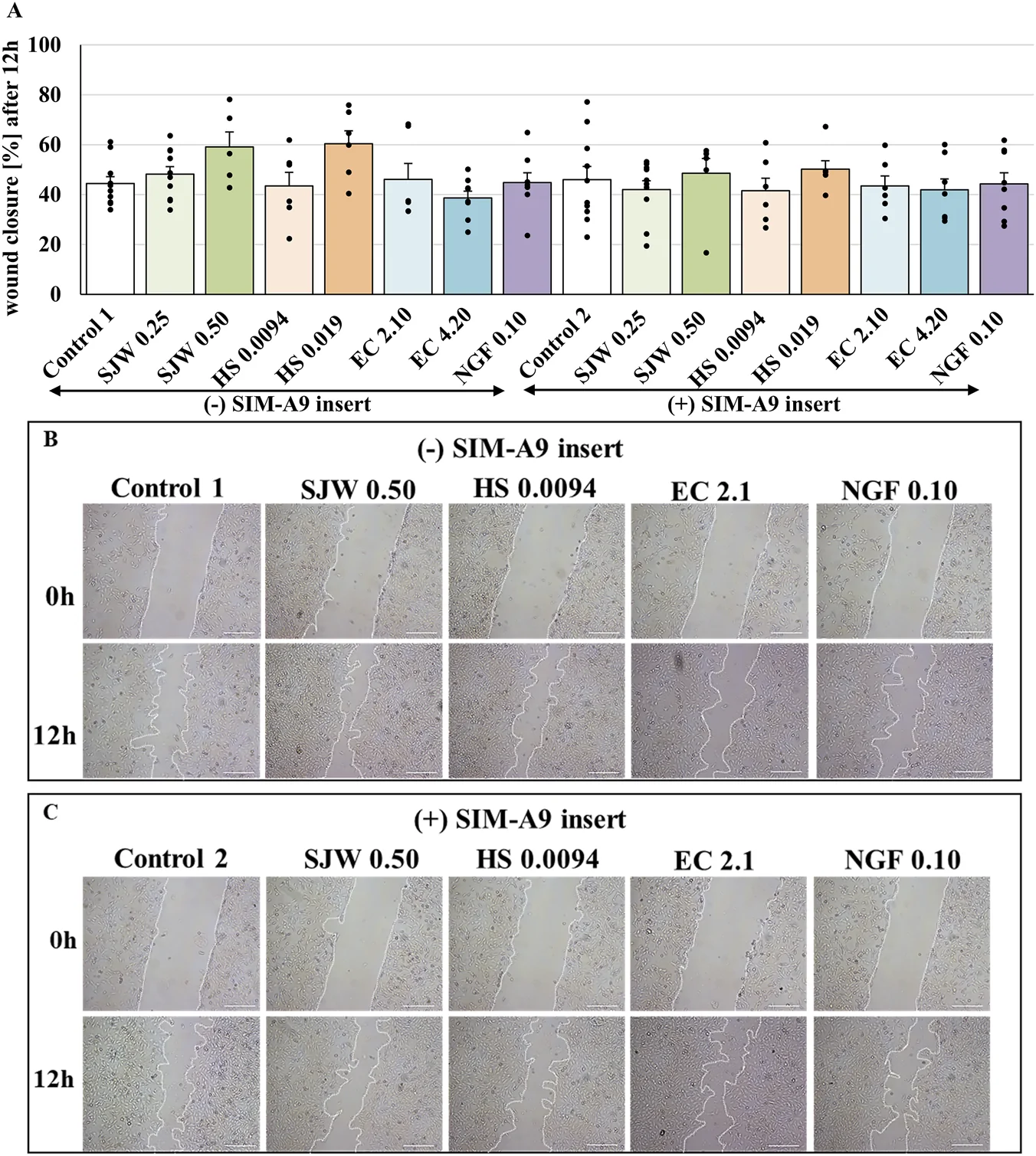
Scratch Assay of mouse hippocampal HT-22 neuronal cells (with or without mouse SIM-A9 microglia) after treatment with SJW, HS, EC, or NGF. **(A)** Quantifying the wound closure in % after 12 h and representative images of the scratches after 0 h and 12 h **(B)** without or **(C)** with mouse SIM-A9 microglia. Data are expressed as mean +SEM; one way ANOVA (Post hoc Dunnett’s method). Scale bar = 1 mm, *n* = 4 independent experiments with 2 replicates per treatment. The substances under test are given in µg/mL.

### 3.6 Effects of SJW, hyperoside, and escitalopram on the paracrine release of protein markers using the ProteomeProfiler™ and ELISA

#### 3.6.1 Cholinergic differentiation factor/leukemia inhibitory factor (CDF/LIF)

To explore paracrine factors beyond BDNF, a known regulator of neuritogenesis (Liu and Nusslock, 2018; Colucci-D'Amato et al., 2020), we conducted a thorough examination of protein markers using the ProteomeProfiler™ screening assay in our co-culture experiments. Treatment of HT-22 co-cultured with SIM-A9 with 0.25 or 0.50 μg/mL SJW increased the release of CDF/LIF by 38.1% and 15.0%, respectively, compared with the respective treatment of HT-22 cultured alone (Figure 8A). Hyperoside at 0.019 μg/mL or escitalopram at 2.10 μg/mL increased the release of CDF/LIF by 46.6% and 33.9%, respectively, compared to the respective treatment of HT-22 cultured alone (Figure 8A). In contrast, HT-22 co-cultured with SIM-A9 showed that 0.10 μg/mL NGF inhibited the release by 20.3% compared with the respective treatment of HT-22 cultured alone (Figure 8A). Moreover, treatment of HT-22 neuronal cells with NGF (0.10 μg/mL) in the absence of SIM-A9 increased the CDF/LIF release by 15.7% compared to control 1 (Figure 8A). In HT-22 in the presence of SIM-A9, SJW at 0.25 or 0.50 μg/mL increased the CDF/LIF release by 40.2% and 21.3%, respectively, compared to control 2 (Figure 8A). Additionally, treatment of HT-22 co-cultured with SIM-A9 with 0.019 μg/mL hyperoside or 2.10 μg/mL escitalopram increased the CDF/LIF release by 40.8% and 38.9%, respectively, compared to control 2, whereas 0.1 μg/mL NGF did not affect the CDF/LIF release (Figure 8A).

**FIGURE 8:**
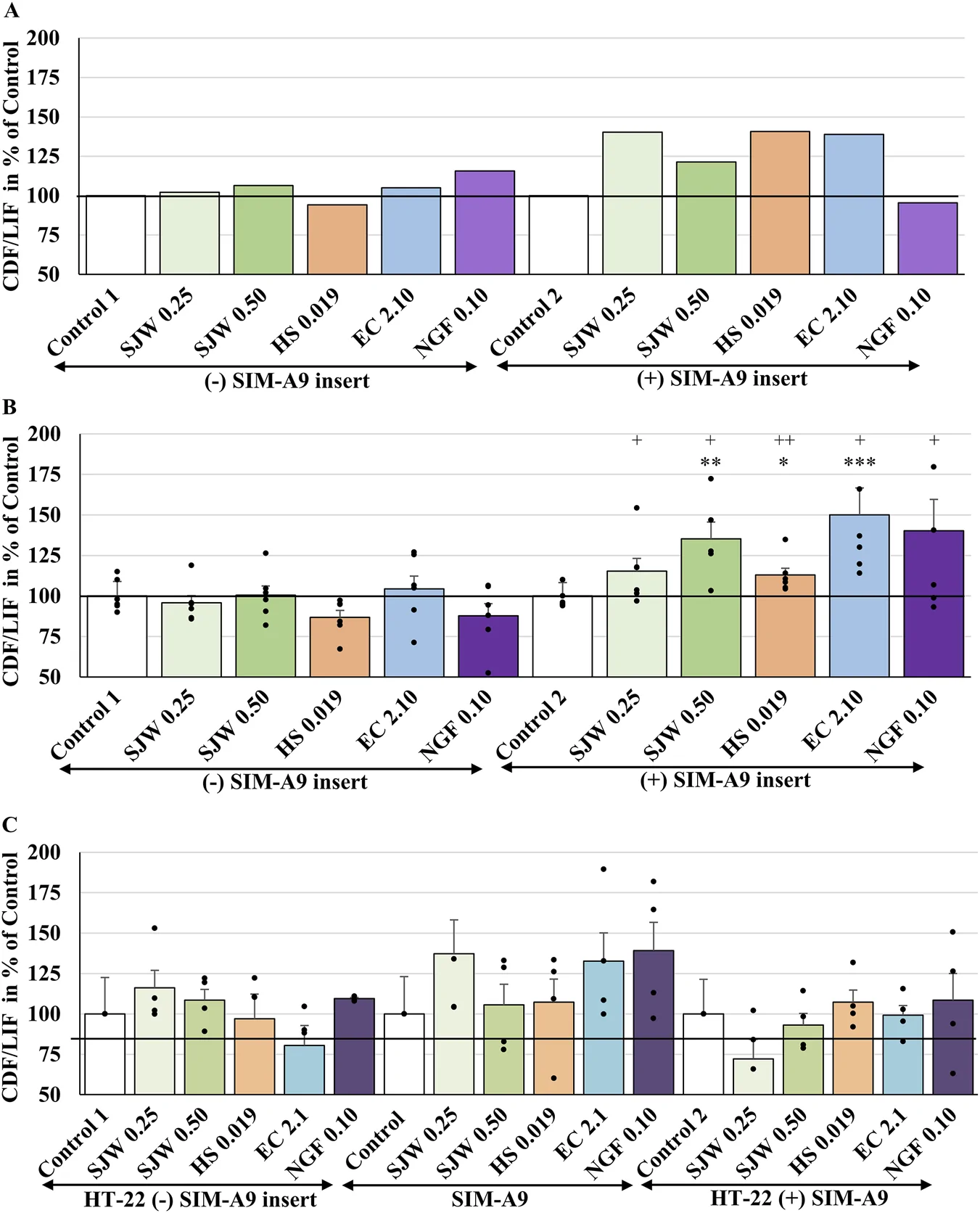
Cholinergic neuronal differentiation factor/leukemia inhibitory factor (CDF/LIF) release of mouse hippocampal HT-22 neuronal cells (with or without mouse SIM-A9 microglia) after 24 h treatment with SJW, HS, EC, or NGF determined in culture medium by **(A)** ProteomeProfiler™ a pool of samples from three experiments, and **(B)** ELISA, *n* = 5 independent experiments with 2 replicates per treatment. Concentrations of CDF/LIF were normalized with the total cell number measured by crystal violet (CV). **(C)** After treatment, cell homogenates from mouse hippocampal HT-22 neuronal cells and mouse SIM-A9 microglia were analyzed by ELISA and normalized to total protein concentration determined by the BCA method. *N* = 5 independent experiments with 2 replicates per treatment. Data from ProteomeProfiler™ are expressed as the mean of pixel intensities from two spots on one array, as a percentage of the control. Data of CDF/LIF ELISA are expressed as mean +SEM; Kruskal-Wallis (*post hoc* Dunn’s method): *p ≤ 0.05, **p ≤ 0.01, ***p ≤ 0.001 significance vs. control 2 or ^+^ *p ≤* 0.05, ^++^ *p ≤* 0.01 vs. respective treatment of mouse hippocampal HT-22 neuronal cells cultured alone. The substances under test are given in µg/mL.

Using the quantitative ELISA method, we found that treatment of HT-22, co-cultured with SIM-A9, with 0.25 or 0.50 μg/mL SJW significantly increased the CDF/LIF release by 19.6% (*p* ≤ 0.041) or 34.7% (*p* ≤ 0.021) compared to the respective treatment of HT-22 cultured alone (Figure 8B). Moreover, treatment of HT-22, co-cultured with SIM-A9 with hyperoside (0.019 μg/mL), escitalopram (2.10 μg/mL) or NGF (0.10 μg/mL) significantly increased the CDF/LIF release by 26.1% (*p* ≤ 0.003), 45.5% (*p* ≤ 0.047) or 52.5% (*p* ≤ 0.041) compared to the respective treatments of HT-22 cultured alone (Figure 8B). Furthermore, HT-22 co-cultured with SIM-A9 treated with 0.50 μg/mL SJW significantly increased CDF/LIF release by 35.2% (*p* ≤ 0.01), whereas 0.25 μg/mL showed a non-significant 15.3% increase (*p* ≤ 0.095), compared to control 2 (Figure 8B). Additionally, treatment of mouse HT-22, co-cultured with SIM-A9 with hyperoside (0.019 μg/mL), escitalopram (2.10 μg/mL) significantly increased the CDF/LIF release by 12.9% (*p* ≤ 0.035), 50.0% (*p* ≤ 0.01) but not significant NGF (0.10 μg/mL) 40.3% (*p* = 0.234) in comparison with control 2 (Figure 8B).

Cell homogenates from HT-22 cultured with/without SIM-A9, as well as homogenates from SIM-A9, were analyzed by ELISA. The values obtained were normalized to the total protein content of the cell homogenates. However, we did not find significant differences between the treatments and their respective controls (Figure 8C).

#### 3.6.2 Interleukin-10 (IL-10)

Pro-inflammatory cytokines impair hippocampal-dependent memory and promote depressive-like behaviors in mice (Yirmiya and Goshen, 2011; Dantzer et al., 2008). The anti-inflammatory cytokine IL-10 promotes learning and memory (Worthen et al., 2020), stimulating its release may have an antidepressant effect.

Treatment of HT-22 co-cultured with SIM-A9 with 0.25 μg/mL or 0.50 μg/mL SJW increased the release of IL-10 by 66.4% and 37.6%, respectively, compared to the respective treatment of HT-22 cultured alone (Figure 9A). Incubation of HT-22 with hyperoside (0.019 μg/mL), escitalopram (2.10 μg/mL), or NGF (0.10 μg/mL) increased IL-10 release by 44.7%, 29.3%, and 15.8%, respectively, compared with the respective treatment without SIM-A9 co-culture (Figure 9A). In the presence of SIM-A9, treatment of HT-22 with 0.25 μg/mL or 0.50 μg/mL SJW increased the release of IL-10 by 88.4% and 38.0%, respectively, compared to the control 2 (Figure 9A). After treatment of HT-22 co-cultured with SIM-A9 with 0.019 μg/mL hyperoside, 2.10 μg/mL escitalopram, or 0.10 μg/mL NGF, the release increased by 84.6%, 60.9% and 39.9%, respectively, compared to control 2 (Figure 9A). Using ELISA, we found that treatment of HT-22, co-cultured with SIM-A9, with 0.25 μg/mL or 0.50 μg/mL SJW increased the IL-10 release by 14.2% or 22.2%, but not significantly (*p* ≤ 0.152 and *p* ≤ 0.159), compared to the respective treatment of HT-22 cultured alone (Figure 9B). In contrast, the treatment of HT-22 with hyperoside (0.019 μg/mL), escitalopram (2.10 μg/mL), or NGF (0.10 μg/mL) increased the IL-10 release significantly by 52.7% (*p* ≤ 0.032), 35.8% (*p* ≤ 0.021), or 54.4% (*p* ≤ 0.026) compared to the respective treatment without SIM-A9 co-culture (Figure 9B). Additionally, treatment with hyperoside (0.019 μg/mL) increased the IL-10 release by 15.6% (*p* ≤ 0.024). Escitalopram (2.10 μg/mL), or NGF (0.10 μg/mL) of HT-22, co-cultured with SIM-A9, significantly (*p* ≤ 0.01) increased the IL-10 release by 24.2% and 35.7% in comparison with control 2 (Figure 9B).

**FIGURE 9:**
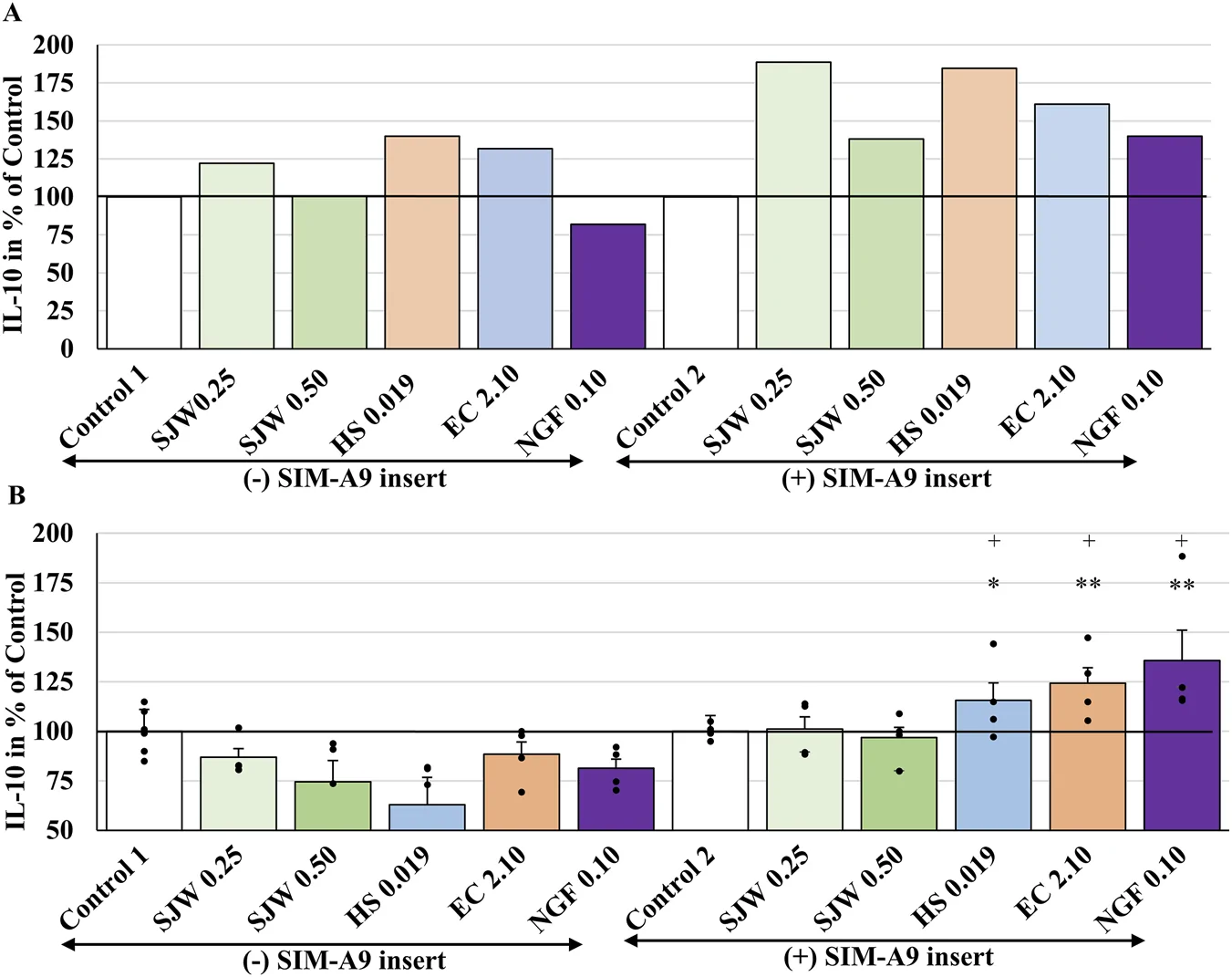
Interleukin 10 (IL-10) release of mouse hippocampal HT-22 neuronal cells (with or without mouse SIM-A9 microglia) after 24 h treatment with SJW, HS, EC, or NGF determined in culture medium by **(A)** ProteomeProfiler™ a pool of samples from three experiments and **(B)** ELISA, *n* = 5 independent experiments with 2 replicates per treatment. Data from ProteomeProfiler™ are expressed as mean of pixels from two spots on one array. Data of IL-10 ELISA are expressed as mean +SEM; Kruskal-Wallis (*post hoc* Dunn’s method): *p ≤ 0.05, **p ≤ 0.01 significance vs. control 2 or ^+^ *p* ≤ 0.05 vs. respective treatment of mouse hippocampal HT-22 neuronal cells cultured alone. The substances under test are given in µg/mL.

#### 3.6.3 Growth/differentiation factor 15 (GDF-15)

GDF-15 is believed to act as a neuroprotective agent in disease and brain injury models; however, its elevation in certain patients, such as those with cardiovascular diseases and cancer, is associated with poorer outcomes (Isik et al., 2024). Consequently, a reduction of GDF-15 release may be interpreted as beneficial (Isik et al., 2024). Interestingly, treatment of HT-22 co-cultured with SIM-A9 with 0.25 μg/mL SJW decreased the GDF-15 release by 29.3% compared to the respective treatment of HT-22 cultured alone (Figure 10A). Moreover, treatment of HT-22 co-cultured with SIM-A9 with 0.019 μg/mL hyperoside decreased GDF-15 release by 29.0%, but not 2.10 μg/mL escitalopram, compared to the respective treatment of HT-22 cultured alone (Figure 10A). Treatment of HT-22 co-cultured with SIM-A9 with 0.10 μg/mL NGF decreased GDF-15 release by 10.9% compared to the respective treatment of HT-22 cultured alone (Figure 10A). Additionally, treatment of HT-22 without SIM-A9 co-culture, with 0.25 μg/mL SJW, 0.019 μg/mL hyperoside, or 0.10 μg/mL NGF increased GDF-15 release by 30.4%, 13.6%, or 33.0% compared to the control 1 (Figure 10A). Interestingly, treatment of co-cultures, with NGF at 0.1 μg/mL increased GDF-15 release by 22.1% compared to the control 2 (Figure 10A). Using ELISA, we found that treatment of HT-22 co-cultured with SIM-A9 with 0.50 μg/mL SJW decreased the GDF-15 release significantly by 16.0% (*p* ≤ 0.010), and 0.019 μg/mL hyperoside inhibited the GDF-15 release significantly by 17.5% (*p* ≤ 0.021) compared to the control 2 (Figure 10B). Cell homogenates from HT-22 cultured with/without SIM-A9, as well as homogenates from SIM-A9, were analyzed by ELISA. Only the cell homogenates from SIM-A9 showed detectable GDF-15 expression (Figure 10C). However, we did not find significant differences among treatments (Figure 10C).

**FIGURE 10:**
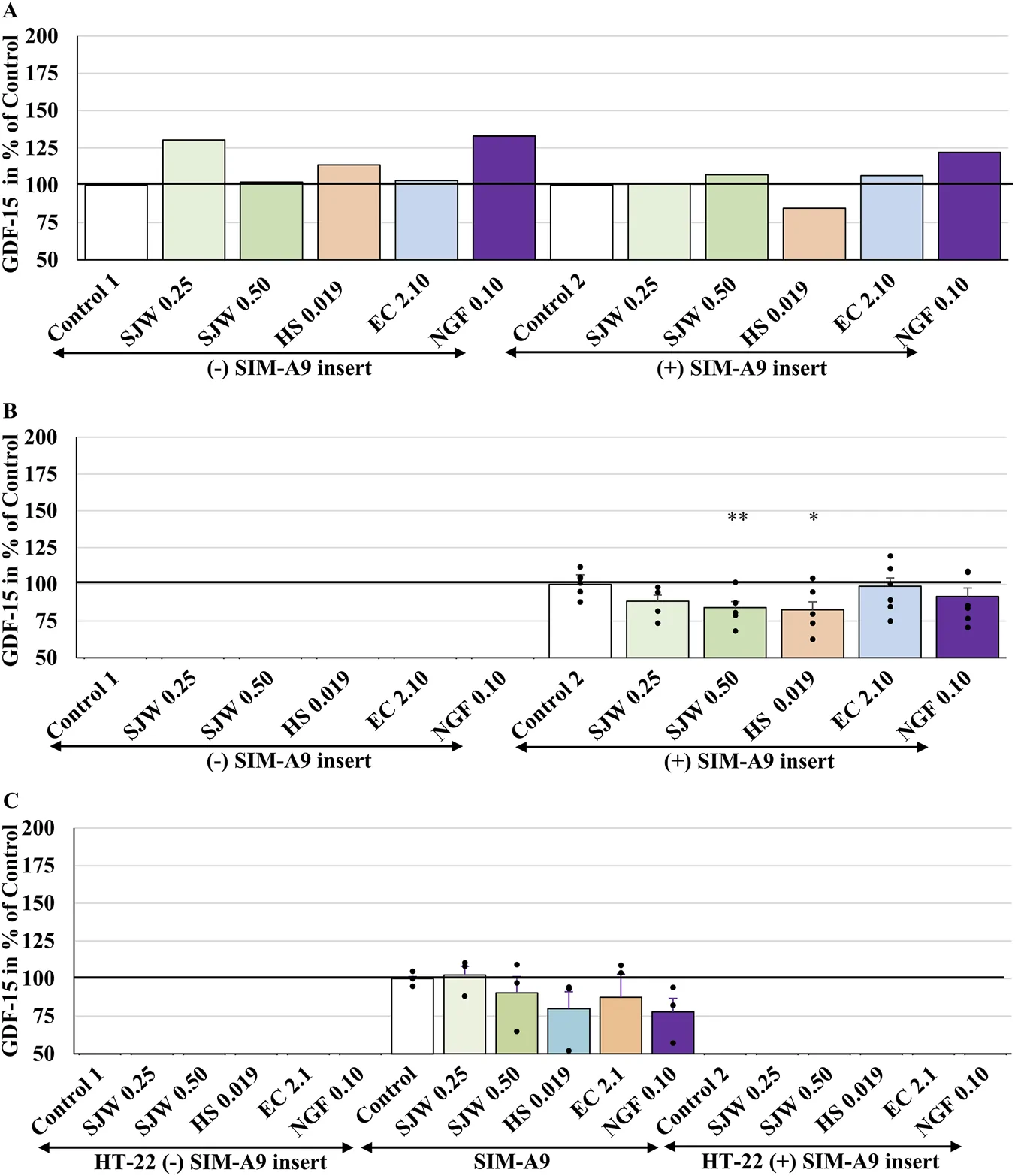
Growth Differentiation Factor-15 (GDF-15) release of mouse hippocampal HT-22 neuronal cells (with or without mouse SIM-A9 microglia) after 24 h treatment with SJW, HS, EC, or NGF determined in culture medium by **(A)** ProteomeProfiler™ a pool of samples from three experiments and **(B)** ELISA, *n* = 4 independent experiments with 2 replicates per treatment. Concentrations of GDF-15 were normalized with the total cell number measured by crystal violet (CV). **(C)** After treatment, cell homogenates of mouse hippocampal HT-22 neuronal cells and mouse SIM-A9 microglia were analyzed by ELISA and normalized to total protein concentration determined by the BCA method, *n* = 3 independent experiments with 2 replicates per treatment. Data from ProteomeProfiler™ are expressed as the mean of pixels from two spots on one array as a percentage of the control. Data from GDF-15 ELISA are expressed as mean +SEM; one way ANOVA (*post hoc* Dunnett’s method): *p ≤ 0.05, **p ≤ 0.01 significance vs. control 2. The substances under test are given in µg/mL.

## 4 Discussion

Natural products that promote neurogenesis and plasticity may help treat depressive states and pathologies, such as cerebral ischemia/reperfusion (I/R) injury, which is associated with neurodegeneration (Farzadmanesh et al., 2025; Li and Wang, 2026). In this context, microglia-inducing trophic functions respond to changes in neural activity (Gras et al., 2012) and to neuronal pruning, thereby maintaining synaptic function during neurogenesis and supporting neuronal plasticity (Schafer and Stevens, 2015; Matcovitch-Natan et al., 2016). To support neuronal plasticity, signaling through the CX3CL1-CX3CR1 axis facilitates communication between healthy neurons and microglia, enabling microglia to protect neuronal integrity (Haidar et al., 2022). CX3CL1 maintains microglia in a neuroprotective state, and disruptions in this signaling dysregulate the microglial phenotype, leading to neuronal damage (Febinger et al., 2015). Additionally, CX3CL1-CX3CR1 signaling is critical for synaptic pruning (Paolicelli et al., 2011; Sakai J., 2020), a process by which microglia eliminate inactive or dysfunctional synapses through phagocytosis, thereby refining synaptic circuits in the adult brain (Sakai J., 2020). In our study, CX3CL1/Fractalkine was included in the ProteomeProfiler screening methods. Still, we observed a low dot signal in the analyzed co-culture medium, with no difference between treatments with or without microglia. Furthermore, we have not experimentally determined whether there are neuron-repairing or pruning synaptic effects. We have previously shown that SJW treatment promotes neurite growth in mouse hippocampal HT-22 neuronal cells, suggesting neurotrophic properties of SJW that may explain its positive effects on depression (Bonaterra et al., 2018). However, unlike our previous publications, neurite formation was actually assessed via fluorometry using a kit rather than through direct microscopic observation; nevertheless, a direct correlation was observed between results of these two methods (Hancock et al., 2015; Broeglin et al., 2026). We have also reported that SJW protects mouse hippocampal HT-22 neuronal cells from glutamate- or NMDA-induced cytotoxicity (Bonaterra et al., 2018). Previously, we showed that SJW exhibits anti-inflammatory and antioxidant properties in microglia and stimulates neurite outgrowth in mouse hippocampal HT-22 neuronal cells, a characteristic of neuronal plasticity and neuritogenesis (Bonaterra et al., 2018; Bonaterra et al., 2020). We hypothesize that the interaction between these 2 cell types might be paracrine. We wish to highlight that we used hyperoside as a control in our experiments because it reversed chronic restraint stress (CRS)-induced depressive-like behaviors in mice (Song et al., 2024). These findings require validation in inflammatory models, organotypic cultures, animal models, and undoubtedly clinical studies. However, it is important to mention that a recent clinical study demonstrates the effectiveness of SJW for the treatment of mild to moderate depression (Kalbermatten and Saller, 2025).

Interestingly, we found stimulation of neurite outgrowth when mouse hippocampal HT-22 cells were co-cultured with mouse SIM-A9 microglia after treatment with the substances under test. A candidate to explain this effect might be BDNF, a key regulator of synaptic plasticity. BDNF is a protein that plays a crucial role in promoting neuronal growth and development and is involved in processes such as neuroplasticity, synaptic modulation, and neuronal survival (Correia et al., 2023). BDNF is implicated in the pathophysiology of disorders such as MDD and psychiatric disorders (Colucci-D’amato et al., 2020; Li et al., 2022; Correia et al., 2023). Stress and consecutive depression reduce BDNF levels, while antidepressant treatment reverses this by raising BDNF levels (Chakrapani et al., 2020).

Furthermore, chronic stress reduces neurogenesis, and antidepressants increase plasticity by enhancing neurogenesis and synaptogenesis in the adult hippocampus (Chakrapani et al., 2020). However, we found that mouse SIM-A9 microglia inhibited BDNF release from HT-22 cells following treatment with the substances. Unlike the literature’s suggestion that higher BDNF levels could promote neuritogenesis, our *in vitro* findings indicate a decrease of the BDNF release. In this context, to investigate, beyond BDNF, the probable origin of the stimulatory effects of the substances under test on neurite outgrowth in a co-culture system of mouse hippocampal HT-22 neuronal cells and mouse SIM-A9 microglia, the release of proteins into the culture medium was analyzed using the screening assay ProteomeProfiler™ with additional confirmation by ELISA. We describe here for the first time that the cholinergic neuronal differentiation factor/leukemia inhibitory factor (CDF/LIF), Growth Differentiation Factor 15 (GDF-15), and the cytokine IL-10 are particularly noteworthy in the context of a possible paracrine interactions. It should be noted that these effects were observed under unstimulated co-culture conditions with basal levels of TNF-α and IL-6 below those reported by others (Nagamoto-Combs et al., 2014; Blank et al., 2022) for the mouse SIM-A9 microglia used in our experiments. In this context, it has been demonstrated that mouse SIM-A9 microglia respond to pro-inflammatory stimuli, such as LPS, and exhibit increased TNF-α release compared to controls under unstimulated conditions without LPS (∼75 pg/mL) (Nagamoto-Combs et al., 2014). In our experiments, basal TNF-α release was 25 pg/mL. It did not increase further after treatment with SJW, hyperoside, escitalopram, or NGF, even when mouse hippocampal HT-22 neuronal cells were co-cultured with mouse SIM-A9 microglia. Similarly, the release of the pro-inflammatory cytokine IL-6 by mouse hippocampal HT-22 neuronal cells was 78.6 pg/mL when co-cultured with mouse SIM-A9 microglia, which is lower than the level induced by pro-inflammatory stimulation with 2.5 ng/mL LPS, which remained below 2000 pg/ml as described by others (Blank et al., 2022). Neuronal plasticity comprises a range of mechanisms, including migration, neurite outgrowth, synaptogenesis, and the maturation of functional synapses (Wainwright and Galea, 2013). We have previously demonstrated that SJW stimulated migration of mouse SIM-A9 microglia (Bonaterra et al., 2020). However, in our present study, we did not detect any stimulation of migration in mouse hippocampal HT-22 neuronal cells, whether co-cultured with microglia or cultured alone, in any treatment, which is unrelated to the observed stimulation of neuritogenesis. Considering that HT-22 neuronal cells have a doubling time of 15 h, the lack of differences in migration following treatment with SJW, hyperoside, escitalopram, or NGF compared to the control may be due to proliferation masking migration. Using proteome analyses, ProteomeProfiler™ and ELISA, we demonstrate for the first time that the production and release of CDF/LIF by mouse hippocampal HT-22 neuronal cells, but only when co-cultured with mouse SIM-A9 microglia, was stimulated, which might be an indication of an effect from SJW on the paracrine pathway. Others have described CDF/LIF as a critical paracrine signaling molecule in the CNS, with key functions in maintaining adult brain health, including neuroprotection and anti-inflammatory effects (Metcalfe, 2019; Lin et al., 2020). CDF/LIF is a cytokine that, among other functions in the nervous system, induces cholinergic differentiation of sympathetic neurons (Moidunny et al., 2012). As already described by other researchers, CDF/LIF is required for overall CNS development and exhibits neuroprotective and neurogenic effects, including myelinogenic and myelin-reparative properties, as well as anti-inflammatory effects (Gao et al., 2009; Metcalfe, 2019). In addition, CDF/LIF plays a crucial role in cognitive and mental health by preserving neuroglial integrity (Metcalfe et al., 2017). Interestingly, CDF/LIF is actively transported across the blood-brain barrier (BBB) and enters the brain and spinal cord, making it a promising candidate for therapeutic use (Pan and Kastin, 2000; Metcalfe et al., 2017). In this therapeutic context, as described by others CDF/LIF administration may act as a neuroprotective and regenerative cytokine following neurodegeneration induced by hypoxic-ischemic encephalopathies or ischemic vessel occlusion (Davis et al., 2017; Lin et al., 2020). The possible beneficial and therapeutic action of CDF/LIF may be mediated by direct effects on neurons and glia or through other mechanisms as paracrine (Lin et al., 2020). It has previously been reported that SJW induces a dual modulation of cholinergic signaling, characterized by both a decrease and an increase in acetylcholine levels, which may improve depressive symptoms (Kholghi et al., 2022). Following brain injury, results support the therapeutic potential of CDF/LIF in reducing neurodegeneration and enhancing neurological outcomes (D'Mello et al., 2025). Additionally, the regenerative capacity of CDF/LIF has been described *in vivo* following closed head injury, frontal cortex injury, and autoimmune demyelination in mice (Linker et al., 2008; Frondelli and Levison, 2021; D'Mello et al., 2025). The observed changes in CDF/LIF suggest a possible role in the effects of SJW that warrants further investigation. Other authors have shown that the CDF/LIF-insulin-like growth factors (IGF) axis participates in cerebral cortex development through autocrine or paracrine mechanisms (Takata et al., 2022) In this context, we hypothesize that our results may support the possible therapeutic use of SJW. However, *in vivo* experiments and clinical studies would be required to validate this hypothesis. Additionally, functional validation experiments are necessary, such as neutralization, gene silencing, receptor inhibition, and conditioned medium transfer, to confirm the paracrine action of CDF/LIF. Moreover, inflammatory cytokines, such as IL-6 and TNF-α, increase the protein and RNA levels of CDF/LIF during the culture of human induced pluripotent stem cells (hiPSC)-derived neural precursor cells (NPCs) (Knight et al., 1999; Palmqvist et al., 2008). However, the increase in CDF/LIF release observed in our present study was not caused by a pro-inflammatory state, as we have demonstrated that the two cytokines, IL-6 and TNF-α, remained at low levels after treatment with any of the substances under test, regardless of whether mouse hippocampal HT-22 neuronal cells were co-cultured with mouse SIM-A9 microglia or not. We hypothesize that our results may indicate that SJW, or its component hyperoside, may support and explain our previously reported anti-inflammatory effects (Bonaterra et al., 2018; Bonaterra et al., 2020) by increasing CDF/LIF. Additional experiments in animal models of depression are needed to confirm this hypothesis.

Another marker related to inflammation and depression, which acts through autocrine/paracrine mechanisms, which has recently been identified, is GDF-15 (Kochlik et al., 2024; Rochette et al., 2021), i.e. elevated blood levels of GDF-15 frequently appear in individuals with chronic psychological stress, anxiety, and depression (Kochlik et al., 2024). Using ProteomeProfiler™ and ELISA, we show that increased GDF-15 production and release by mouse SIM-A9 microglia treated with SJW may indicate a possible role for GDF-15 in a paracrine pathway influenced by SJW. Nonetheless, *in vivo* experiments and clinical trials are necessary to validate the potential antidepressant effects of GDF-15 and CDF/LIF. It is worth noting that ELISA analysis of cell extracts showed that GDF-15 was detected in mouse SIM-A9 microglia but not in mouse hippocampal HT-22 neuronal cells, while CDF/LIF was present in both cell types. The expression of GDF-15 has been previously reported in microglia (Schober et al., 2001). However, GDF-15 has also been described in neurons (Strelau et al., 2000). Neurodegeneration induces GDF-15 in proportion to disease severity as a healing response (Breit et al., 2021; Tsai et al., 2018). However, the chronic elevation of GDF-15 eventually proves inadequate to alleviate or reverse disease-related stress (Breit et al., 2021; Tsai et al., 2018; Kumar et al., 2017). High serum GDF-15 levels in patients with depression may be associated with cognitive frailty and depression, an increased risk of suicidal ideation and deteriorated cognitive performance (Fuchs et al., 2013; Peng et al., 2021; Kochlik et al., 2024). By contrast, GDF-15 deficiency may have beneficial effects on symptoms associated with induced depression in mice, as evidenced by increased fear-associated memory, sensorimotor improvement, and improved cognition (Low et al., 2017). Additionally, it has been reported that neutralizing GDF-15 in the CNS alleviated pro-inflammatory microglial activation induced after the engulfment of superfluous synapses, thereby reducing inflammation-induced cognitive and memory decline (Chen et al., 2025). GDF15, a soluble factor that performs complex biological functions through autocrine or paracrine pathways, can act on microglial cells to increase TGFβRII expression (Bauskin et al., 2000; Wang et al., 2019). In this context, we hypothesize that a decrease in GDF-15 levels, as shown here for the first time, could be a desirable objective for a potential antidepressant strategy. To validate this hypothesis, additional *in vitro* and animal models, as well as clinical studies, are needed. In any case, the main limitations of our CDF/LIF and GDF-15 findings, obtained only in a cell culture model using cell lines such as HT-22 neuronal cells and SIM-A9 microglia, are not sufficient to claim that the results are translatable to clinical cases of depression, because complementary *in vitro* and *in vivo* experiments, as well as appropriate clinical studies, are additionally required. According to our results, we hypothesize that the interaction between these 2 cell types may be paracrine. However, future functional validation experiments are necessary, such as neutralization, gene silencing, receptor inhibition, and conditioned medium transfer, to confirm our hypothesized the paracrine action. Therefore, in this study, we show for the first time the effects of SJW, hyperoside, and escitalopram on mouse hippocampal HT-22 neuronal cells and mouse SIM-A9 microglia. These findings require validation in inflammatory models, organotypic cultures, animal models, and, undoubtedly, clinical studies.

It should also be noted that quantifying the amount of cytokines released into the co-culture medium presents a significant limitation. Because the doubling rates of HT-22 and SIM-A9 cells differ, it is not possible to accurately predict the number of cells of each type present at the end of the experiment, which can influence the quantity of cytokines released into the culture medium. Nevertheless, by keeping the initial cell count, as well as the incubation and co-incubation conditions and durations, constant, it is possible to obtain comparable results for analyzing the paracrine release of the factors under study.

SJW and its main active components cross the blood-brain barrier poorly or in very limited amounts (Caccia and Gobbi, 2009), though some constituents may still exert indirect or peripheral effects that influence the central nervous system. However, it is not possible to relate concentrations of SJW used *in vitro* (Bonaterra et al., 2018; Bonaterra et al., 2020) to the doses used *in vivo* or in clinical studies (Kalbermatten and Saller, 2025). The relationship between concentrations used via direct *in vitro* exposure and the systemic or cerebral exposures attainable after oral administration of SJW is limited by the poor penetration of its components across the blood-brain barrier (Kalbermatten and Saller, 2025). It is possible that, rather than acting directly on hippocampal neurons and microglia, SJW’s effect may be mediated by systemic activation of other factors that can cross the blood-brain barrier and modulate the cellular milieu in the CNS. Direct paracrine activation of neurons and microglia *in vitro* co-cultures only approximates the effects observed *in vivo* and in clinical studies and is not entirely comparable. Additional pharmacokinetic data on the brain concentrations of these and other constituents and their metabolites are therefore required to more meaningfully interpret the central effects of SJW extract. In this context, Escitalopram, a selective SSRIs, and Hyperoside, a compound found in SJW, influence neurotransmitters such as serotonin and dopamine and can cross the blood-brain barrier (Fornal et al., 2001; Dionisie et al., 2021; Song et al., 2024; Zhang et al., 2025). SJW can interfere with the metabolism of various medications, including oral contraceptives, cancer treatments, HIV antiretrovirals, and antidepressants (Maldonado-Puebla et al., 2025). Combining SJW with other antidepressants may lead to adverse effects. In this context, it can significantly affect the metabolism of SSRIs and raise the risk of serotonin syndrome when taken with SSRIs like escitalopram, which can be serious and potentially life-threatening (Maldonado-Puebla et al., 2025). Additional research is needed to determine the risk profile of SSRIs when combined with SJW. However, using Escitalopram and Hyperoside is suitable for studying potential paracrine effects *in vitro*, which may also translate to *in vivo* CNS mechanisms.

## 5 Conclusion

Our data show that, under unstimulated co-culture conditions, an increase in CDF/LIF levels in HT-22 cells in the presence of SIM-A9 cells, a reduction in GDF-15 release by SIM-A9 microglia, and stimulation of neurite growth. Whereas IL-10 changes were more clearly supported by hyperoside, escitalopram, and NGF than by SJW itself. This interaction between HT-22 hippocampal-derived cells and SIM-A9 microglia may be paracrine. Still, additional functional validation, including *in vitro* and *in vivo* experiments, are necessary to confirm this hypothesis and its association with depressive states. It is reasonable to assume that these characteristics could also serve as beneficial therapeutic targets in the treatment of patients with depressive disorders.

## 6 Limitations of the study

1. The use of a cell culture model using cell lines such as HT-22 neuronal cells and SIM-A9 microglia, rather than primary cells.
2. Additionally, functional validation experiments are necessary, such as neutralization, gene silencing, receptor inhibition, and conditioned medium transfer, to confirm the paracrine action.
3. Interpretation as a paracrine mechanism is limited due to the use of co-culture transwell inserts, because no direct contact between HT-22 and SIM-A9 microglia avoids the study of pruning and phagocytosis in the co-culture and consequently how it affects the results.
4. Soluble factors released by either cell type dilute quickly into the large media volume of the upper and lower wells.
5. The soluble factors released by either cell type are diluted in the medium of the upper and lower wells, making it impossible to determine with certainty which cell type releases them.
6. Both cell types must survive in a single culture medium that may prove suboptimal for the metabolic needs of one of them specifically.
7. Further *in vivo* experiments and clinical studies are required to continue validating the antidepressant effect of SJW.
8. An anti-inflammatory effect cannot be claimed, as no experiments were conducted in the presence of pro-inflammatory factors such as LPS.
9. Proteomic analysis was performed from a pooled sample of each treatment, which does not allow the observation of differences between the replicates.
10. The concentrations of the tested substances were selected based on viability and cytotoxicity data rather than on their effects on tissues or organisms; this represents a limitation regarding the interpretation of our results and the assessment of any potential antidepressant effect.

## Data Availability

The original contributions presented in the study are included in the article/Supplementary Material, further inquiries can be directed to the corresponding author.

## References

[B1] Abdel-Aziz H. Schneider M. Neuhuber W. Müller J. Meguid Kassem A. 2016). GPR84 and TREM-1 signaling contribute to the pathogenesis of Reflux Esophagitis. Mol. Med. 21(1),1011–1024. 10.2119/molmed.2015.00098 26650186 PMC4982478

[B2] Andreska T. Aufmkolk S. Sauer M. Blum R. (2014). High abundance of BDNF within glutamatergic presynapses of cultured hippocampal neurons. Front. Cell. Neurosci. 8, 107. 10.3389/fncel.2014.00107 24782711 PMC3990111

[B3] Bauskin A. R. Zhang H. P. Fairlie W. D. He X. Y. Russell P. K. Moore A. G. (2000). The propeptide of macrophage inhibitory cytokine (MIC-1), a TGF-betasuperfamily member, acts as a quality control determinant forcorrectly folded MIC-1. EMBO J. 19, 2212–2220. 10.1093/emboj/19.10.2212 10811612 PMC384362

[B4] Björkholm C. Monteggia L. M. (2016). BDNF - a key transducer of anti-depressant effects. Neuropharmacology 02, 72–79. 10.1016/j.neuropharm.2015.10.034 26519901 PMC4763983

[B5] Blank M. Enzlein T. Hopf C. (2022). LPS-induced lipid alterations in microglia revealed by MALDI mass spectrometry-based cell fingerprinting in neuroinflammation studies. Sci. Rep. 12 (1), 2908. 10.1038/s41598-022-06894-1 35190595 PMC8861089

[B6] Bonaterra G. A. Schwendler A. Hüther J. Schwarzbach H. Schwarz A. Kolb C. (2018). Neurotrophic, cytoprotective, and anti-inflammatory effects of st. John’s wort extract on differentiated mouse hippocampal HT-22 neurons. Front. Pharmacol. 8, 955. 10.3389/fphar.2017.00955 29403374 PMC5778116

[B7] Bonaterra G. A. Mierau O. Hofmann J. Schwarzbach H. Aziz-Kalbhenn H. Kolb C. (2020). *In vitro* effects of St. John's wort extract against inflammatory and oxidative stress and in the phagocytic and migratory activity of mouse SIM-A9 microglia. Front. Pharmacol. 11, 603575. 10.3389/fphar.2020.603575 33628177 PMC7898673

[B8] Breit S. N. Brown D. A. Tsai V. W. (2021). The GDF15-GFRAL pathway in health and Metabolic disease: friend or foe? Annu. Rev. Physiol. 83, 127–151. 10.1146/annurev-physiol-022020-045449 33228454

[B9] Breyer A. Elstner M. Gillessen T. Weiser D. Elstner E. (2007). Glutamate-induced cell death in neuronal HT22 cells is attenuated by extracts from st. John's wort (Hypericum perforatum L.). Phytomedicine 14 (4), 250–255. 10.1016/j.phymed.2007.02.001 17346956

[B10] Brioschi S. d'Errico P. Amann L. S. Janova H. Wojcik S. M. Meyer-Luehmann M. (2020). Detection of synaptic proteins in Microglia by flow cytometry. Front. Mol. Neurosci. 13, 149. 10.3389/fnmol.2020.00149 33132837 PMC7550663

[B11] Broeglin A. Buetzberger O. Mongeon R. Riou A. Eckert A. Grimm A. (2026). Protocol for automated quantification of neuronal cells using the Agilent BioTek Cytation system and Gen5 neurite outgrowth module. Star. Protoc. 7 (2), 104485. 10.1016/j.xpro.2026.104485 41926289 PMC13068831

[B12] Caccia S. Gobbi M. (2009). St. John's wort components and the brain: uptake, concentrations reached and the mechanisms underlying pharmacological effects. Curr. Drug Metab. 10 (9), 1055–1065. 10.2174/138920009790711878 20214594

[B13] Chakrapani S. Eskander N. De Los Santos L. A. Omisore B. A. Mostafa J. A. (2020). Neuroplasticity and the biological role of brain derived neurotrophic factor in the pathophysiology and management of depression. Cureus 12 (11), e11396. 10.7759/cureus.11396 33312794 PMC7725195

[B14] Chen T. R. (1977). *In situ* detection of mycoplasma contamination in cell cultures by fluorescent Hoechst 33258 stain. Exp. Cell Res. 104 (2), 255–262. 10.1016/0014-4827(77)90089-1 65285

[B15] Chen L. Luo S. Liu T. Shuai Z. Song Y. Yang Q. (2025). Growth differentiation factor 15 aggravates sepsis-induced cognitive and memory impairments by promoting microglial inflammatory responses and phagocytosis. J. Neuroinflammation 22 (1), 44. 10.1186/s12974-025-03369-8 39985040 PMC11846340

[B16] Colucci-D'Amato L. Speranza L. Volpicelli F. (2020). Neurotrophic factor BDNF, physiological functions and therapeutic potential in depression, neurodegeneration and. Brain Cancer. Int. J. Mol. Sci. 21 (20), 7777. 10.3390/ijms21207777 PMC7589016 33096634

[B17] Cornell J. Salinas S. Huang H. Y. Zhou M. (2022). Microglia regulation of synaptic plasticity and learning and memory. Neural. Regen. Res. 17 (4), 705–716. 10.4103/1673-5374.322423 34472455 PMC8530121

[B18] Correia A. S. Cardoso A. Vale N. (2023). BDNF unveiled: exploring its role in major depression disorder serotonergic imbalance and associated stress conditions. Pharmaceutics 15 (8), 2081. 10.3390/pharmaceutics15082081 37631295 PMC10457827

[B19] D'Mello V. Mihailovic J. Ali S. Sanganahalli B. G. Coman D. Hyder F. (2025). Leukemia Inhibitory Factor as a late-stage treatment for delayed white matter loss in concussive head injury. bioRxiv 7, 2025. [Preprint]. 10.1101/2025.04.07.647435

[B20] Dantzer R. O'Connor J. C. Freund G. G. Johnson R. W. Kelley K. W. (2008). From inflammation to sickness and depression: when the immune system subjugates the brain. Nat. Rev. Neurosci. 9 (1), 46–56. 10.1038/nrn2297 PMC2919277 18073775

[B21] Dauncey E. A. Irving J. T. W. Allkin R. A. (2019). A review of issues of nomenclature and taxonomy of Hypericum perforatum L. and Kew's Medicinal Plant Names Services. J. Pharm. Pharmacol. 71 (1), 4–14. 10.1111/jphp.12831 29034955 PMC6585798

[B22] Davis S. M. Pennypacker K. R. (2018). The role of the leukemia inhibitory factor receptor in neuroprotective signaling. Pharmacol. Ther. 183, 50–57. 10.1016/j.pharmthera.2017.08.008 28827150 PMC6265657

[B23] Davis S. M. Collier L. A. Leonardo C. C. Seifert H. A. Ajmo C. T. Jr Pennypacker K. R. (2017). Leukemia inhibitory factor protects neurons from ischemic damage *via* upregulation of superoxide dismutase 3. Mol. Neurobiol. 54 (1), 608–622. 10.1007/s12035-015-9587-2 26746670 PMC5026633

[B24] Deng S. L. Chen J. G. Wang F. (2020). Microglia: a central player in depression. Curr. Med. Sci. 40 (3), 391–400. 10.1007/s11596-020-2193-1 32681244

[B25] Dionisie V. Ciobanu A. M. Toma V. A. Manea M. C. Baldea I. Olteanu D. (2021). Escitalopram targets oxidative stress, Caspase-3, BDNF and MeCP2 in the Hippocampus and frontal cortex of a rat model of depression induced by chronic unpredictable mild stress. Int. J. Mol. Sci. 22 (14), 7483. 10.3390/ijms22147483 34299103 PMC8304451

[B91] European Pharmacopeia (2008). 6th Edn, Supplement 6.2. Pharmeuropa Vol. 20, No. 1, January 2008. Available on at: 193.164.228.37/en/EDQMDownloads-527.html#1000 .

[B26] Fan H. H. Zhu L. B. Li T. Zhu H. Wang Y. N. Ren X. L. (2017). Hyperoside inhibits lipopolysaccharide-induced inflammatory responses in microglial cells *via* p38 and NFκB pathways. Int. Immunopharmacol. 50, 14–21. 10.1016/j.intimp.2017.06.004 28622577

[B27] Farzadmanesh H. Sameni H. Ghanbari A. Vafaei A. A. Khaleghian A. Mirmohammadkhani M. (2025). Preventing neurons and glial cells destruction in Substantia Nigra Pars Compacta and striatum using St. John's wort and stem cells on parkinson's model. Brain Behav. 15 (12), e71124. 10.1002/brb3.71124 41355350 PMC12682933

[B28] Febinger H. Y. Thomasy H. E. Pavlova M. N. Ringgold K. M. Barf P. R. George A. M. (2015). Time-dependent effects of CX3CR1 in a mouse model of mild traumatic brain injury. J. Neuroinflammation 12, 154. 10.1186/s12974-015-0386-5 26329692 PMC4557842

[B29] Ferrini F. De Koninck Y. (2013). Microglia control neuronal network excitability *via* BDNF signalling. Neural. Plast. 2013, 429815. 10.1155/2013/429815 24089642 PMC3780625

[B30] Fornal C. A. Metzler C. W. Mirescu C. Stein S. K. Jacobs B. L. (2001). Effects of standardized extracts of st. John's wort on the single-unit activity of serotonergic dorsal Raphe neurons in awake cats: comparisons with fluoxetine and sertraline. Neuropsychopharmacology 25 (6), 858–870. 10.1016/S0893-133X(01)00297-4 11750179

[B31] Frondelli M. J. Levison S. W. (2021). Leukemia inhibitory factor is required for Subventricular Zone astrocyte progenitor proliferation and for Prokineticin-2 production after a closed head injury in mice. Neurotrauma Rep. 2 (1), 285–302. 10.1089/neur.2020.0063 34223558 PMC8244521

[B32] Fuchs T. Trollor J. N. Crawford J. Brown D. A. Baune B. T. Samaras K. (2013). Macrophage inhibitory cytokine-1 is associated with cognitive impairment and predicts cognitive decline - the Sydney Memory and Aging Study. Aging Cell 12 (5), 882–889. 10.1111/acel.12116 23758647

[B33] Gao W. Thompson L. Zhou Q. Putheti P. Fahmy T. M. Strom T. B. (2009). Treg *versus* Th17 lymphocyte lineages are cross-regulated by LIF *versus* IL-6. Cell Cycle 8 (9), 1444–1450. 10.4161/cc.8.9.8348 19342884 PMC2881570

[B34] Gastpar M. Singer A. Zeller K. (2006). Comparative efficacy and safety of a once-daily dosage of hypericum extract STW3-VI and citalopram in patients with moderate depression: a double-blind, randomised, multicentre, placebo-controlled study. Pharmacopsychiatry 39 (2), 66–75. 10.1055/s-2006-931544 16555167

[B35] Gras G. Samah B. Hubert A. Leone C. Porcheray F. Rimaniol A. C. (2012). EAAT expression by macrophages and microglia: still more questions than answers. Amino Acids 42, 221–229. 10.1007/s00726-011-0866-6 21373769

[B90] Grundmann O. Lv Y. Kelber O. Butterweck V. (2010). Mechanism of St. Johns wort extract (STW3-VI) during chronic restraint stress is mediated by the interrelationship of the immune, oxidative defense, and neuroendocrine system. Neuropharmacology 58, 767–773. 10.1016/j.neuropharm.2009.12.014 20036263

[B36] Haidar M. A. Ibeh S. Shakkour Z. Reslan M. A. Nwaiwu J. Moqidem Y. A. (2022). Crosstalk between microglia and neurons in Neurotrauma: an overview of the underlying mechanisms. Curr. Neuropharmacol. 20 (11), 2050–2065. 10.2174/1570159X19666211202123322 34856905 PMC9886840

[B37] Hancock M. K. Kopp L. Kaur N. Hanson B. J. (2015). A facile method for simultaneously measuring neuronal cell viability and neurite outgrowth. Curr. Chem. Genom Transl. Med. 9, 6–16. 10.2174/2213988501509010006 PMC4382562 25853055

[B38] Hellwig S. Brioschi S. Dieni S. Frings L. Masuch A. Blank T. (2016). Altered microglia morphology and higher resilience to stress-induced depression-like behavior in CX3CR1-deficient mice. Brain Behav. Immun. 55, 126–137. 10.1016/j.bbi.2015.11.008 26576722

[B39] Hofrichter J. Krohn M. Schumacher T. Lange C. Feistel B. Walbroel B. (2013). Reduced Alzheimer's disease pathology by st. John's Wort treatment is independent of hyperforin and facilitated by ABCC1 and microglia activation in mice. Curr. Alzheimer Res. 10 (10), 1057–1069. 10.2174/15672050113106660171 24156265 PMC3909922

[B40] Huang J. Zhou L. Chen J. Chen T. Lei B. Zheng N. (2021). Hyperoside attenuate inflammation in HT22 cells *via* upregulating SIRT1 to activities Wnt/β-Catenin and sonic Hedgehog pathways. Neural Plast. 2021, 8706400–8706410. 10.1155/2021/8706400 34221003 PMC8213468

[B41] Isik F. I. Thomson S. Cueto J. F. Spathos J. Breit S. N. Tsai V. W. W. (2024). A systematic review of the neuroprotective role and biomarker potential of GDF15 in neurodegeneration. Front. Immunol. 15, 1514518. 10.3389/fimmu.2024.1514518 39737171 PMC11682991

[B42] Jesulola E. Micalos P. Baguley I. J. (2018). Understanding the pathophysiology of depression: from monoamines to the neurogenesis hypothesis model - are we there yet? Behav. Brain Res. 341, 79–90. 10.1016/j.bbr.2017.12.025 29284108

[B991] Jungke P. Ostrow G. Li J. L. Norton S. Nieber K. Kelber O. (2011). Profiling of hypothalamic and hippocampal gene expression in chronically stressed rats treated with St. Johns wort extract (STW 3-VI) and fluoxetine. Psychopharmacology 213, 757–772. 10.1007/s00213-010-2032-3 20924750

[B43] Kalbermatten N. Saller R. (2025). St John's wort (Hypericum perforatum) fresh plant tincture for patients with mild to moderate depression: a prospective observational Study. Complement. Med. Res. 32 (5), 363–375. 10.1159/000547920 40906611 PMC12622987

[B44] Kholghi G. Arjmandi-Rad S. Zarrindast M. R. Vaseghi S. (2022). St. John's wort (Hypericum perforatum) and depression: what happens to the neurotransmitter systems? Naunyn Schmiedeb. Arch. Pharmacol. 395 (6), 629–642. 10.1007/s00210-022-02229-z 35294606

[B45] Knight D. A. Lydell C. P. Zhou D. Weir T. D. Schellenberg R. Bai T. R. (1999). Leukemia inhibitory factor (LIF) and LIF receptor in human lung. Distribution and regulation of LIF release. Am. J. Respir. Cell Mol. Biol. 20, (4), 834–841. 10.1165/ajrcmb.20.4.3429 10101017

[B46] Kochlik B. Herpich C. Moreno-Villanueva M. Klaus S. Müller-Werdan U. Weinberger B. (2024). Associations of circulating GDF15 with combined cognitive frailty and depression in older adults of the MARK-AGE study. Geroscience 46 (2), 1657–1669. 10.1007/s11357-023-00902-6 37715843 PMC10828354

[B47] Kumar P. Millischer V. Villaescusa J. C. Nilsson I. A. K. Östenson C. G. Schalling M. (2017). Plasma GDF15 level is elevated in psychosis and inversely correlated with severity. Sci. Rep. 7 (1), 7906. 10.1038/s41598-017-07503-2 28801589 PMC5554200

[B48] Kwon S. H. Han J. K. Choi M. Kwon Y. J. Kim S. J. Yi E. H. (2017). Dysfunction of microglial STAT3 alleviates depressive behavior *via* Neuron-Microglia interactions. Neuropsychopharmacology 42 (10), 2072–2086. 10.1038/npp.2017.93 28480882 PMC5561349

[B89] Lazaratou H. Dikeos D. G. Anagnostopoulos D. C. Soldatos C. R. (2010). Depressive symptomatology in high school students: the role of age, gender and academic pressure. Community Ment. Health J. 46 (3), 289–295. 10.1007/s10597-010-9296-2a 20140753

[B49] Li T. Wang C. (2026). Hypericin alleviates cerebral ischemia/reperfusion injury by modulating endoplasmic reticulum stress. Front. Pharmacol. 16, 1723495. 10.3389/fphar.2025.1723495 41573713 PMC12819193

[B50] Li Y. Li F. Qin D. Chen H. Wang J. Wang J. (2022). The role of brain derived neurotrophic factor in central nervous system. Front. Aging Neurosci. 14, 986443. 10.3389/fnagi.2022.986443 36158555 PMC9493475

[B51] Lin J. Niimi Y. Clausi M. G. Kanal H. D. Levison S. W. (2020). Neuroregenerative and protective functions of Leukemia Inhibitory Factor in perinatal hypoxic-ischemic brain injury. Exp. Neurol. 330, 113324. 10.1016/j.expneurol.2020.113324 32320698 PMC7304440

[B52] Linker R. A. Kruse N. Israel S. Wei T. Seubert S. Hombach A. (2008). Leukemia inhibitory factor deficiency modulates the immune response and limits autoimmune demyelination: a new role for neurotrophic cytokines in neuroinflammation. J. Immunol. 180 (4), 2204–2213. 10.4049/jimmunol.180.4.2204 18250427

[B53] Liu P. Z. Nusslock R. (2018). Exercise-Mediated neurogenesis in the Hippocampus *via* BDNF. Front. Neurosci. 12, 52. 10.3389/fnins.2018.00052 29467613 PMC5808288

[B54] Liu W. Ge T. Leng Y. Pan Z. Fan J. Yang W. (2017). The role of neural plasticity in depression: from Hippocampus to prefrontal cortex. Neural Plast. 2017, 6871089. 10.1155/2017/6871089 28246558 PMC5299163

[B55] Liu Q. He H. Yang J. Feng X. Zhao F. Lyu J. (2020). Changes in the global burden of depression from 1990 to 2017: findings from the Global Burden of Disease study. J. Psychiatr. Res. 126, 134–140. 10.1016/j.jpsychires.2019.08.002 31439359

[B56] Liu Y. J. Spangenberg E. E. Tang B. Holmes T. C. Green K. N. Xu X. (2021). Microglia elimination increases neural circuit connectivity and activity in adult Mouse cortex. J. Neurosci. 41 (6), 1274–1287. 10.1523/JNEUROSCI.2140-20.2020 33380470 PMC7888230

[B57] Low J. K. Ambikairajah A. Shang K. Brown D. A. Tsai V. W. Breit S. N. (2017). First behavioural characterisation of a knockout mouse model for the transforming growth factor (TGF)-β superfamily cytokine, MIC-1/GDF15. PLoS One 12 (1), e0168416. 10.1371/journal.pone.0168416 28081177 PMC5231335

[B58] Maldonado-Puebla R. A. Choudhury N. Carr B. (2025). The effects of St. John’s wort and its interactions with SSRI’s. Eur. Psychiatry 68 (S1), S1031. 10.1192/j.eurpsy.2025.2087

[B59] Matcovitch-Natan O. Winter D. R. Giladi A. Vargas Aguilar S. Spinrad A. Sarrazin S. (2016). Microglia development follows a stepwise program to regulate brain homeostasis. Science 353 (6301), aad8670. 10.1126/science.aad8670 27338705

[B60] Metcalfe S. M. (2019). Neuroprotective Immunity: leukaemia Inhibitory Factor (LIF) as guardian of brain health. Med. Drug Discov. 2, 100006. 10.1016/j.medidd.2019.100006

[B61] Metcalfe S. M. Bickerton S. Fahmy T. (2017). Neurodegenerative disease: a perspective on cell-based therapy in the new era of cell-free nano-therapy. Curr. Pharm. Des. 23 (5), 776–783. 10.2174/1381612822666161206141744 27924726 PMC5662026

[B62] Moidunny S. Vinet J. Wesseling E. Bijzet J. Shieh C. H. van Ijzendoorn S. C. (2012). Adenosine A2B receptor-mediated leukemia inhibitory factor release from astrocytes protects cortical neurons against excitotoxicity. J. Neuroinflammation 9, 198. 10.1186/1742-2094-9-198 22894638 PMC3458985

[B63] Nagamoto-Combs K. Kulas J. Combs C. K. (2014). A novel cell line from spontaneously immortalized murine microglia. J. Neurosci. Methods 233, 187–198. 10.1016/j.jneumeth.2014.05.021 24975292 PMC4140094

[B64] Palmqvist P. Lundberg P. Lundgren I. Hänström L. Lerner U. H. (2008). IL-1beta and TNF-alpha regulate IL-6-type cytokines in gingival fibroblasts. J. Dent. Res. 87 (6), 558–563. 10.1177/154405910808700614 18502965

[B65] Pan W. Kastin A. J. (2000). Brennan JM. Saturable entry of leukemia inhibitory factor from blood to the central nervous system. J. Neuroimmunol. 106 (1-2), 172–180. 10.1016/s0165-5728(00)00241-1 10814795

[B66] Paolicelli R. C. Bolasco G. Pagani F. Maggi L. Scianni M. Panzanelli P. (2011). Synaptic pruning by microglia is necessary for normal brain development. Science 333 (6048), 1456–1458. 10.1126/science.1202529 21778362

[B67] Parkhurst C. N. Yang G. Ninan I. Savas J. N. Yates J. R. 3rd Lafaille J. J. (2013). Microglia promote learning-dependent synapse formation through brain-derived neurotrophic factor. Cell 155 (7), 1596–1609. 10.1016/j.cell.2013.11.030 24360280 PMC4033691

[B68] Peng R. Li D. Mei S. Q. Li Y. (2021). The Association among serum growth differentiation factor 15 level and suicidal ideation is dependent on testosterone deficiency in depressive patients. J. Inflamm. Res. 14, 2723–2730. 10.2147/JIR.S313200 34194235 PMC8238534

[B69] Pollak D. D. Rey C. E. Monje F. J. (2010). Rodent models in depression research: classical strategies and new directions. Ann. Med. 42 (4), 252–264. 10.3109/07853891003769957 20367120

[B70] Rochette L. Dogon G. Zeller M. Cottin Y. Vergely C. (2021). GDF15 and cardiac cells: current concepts and new insights. Int. J. Mol. Sci. 22 (16), 8889. 10.3390/ijms22168889 34445593 PMC8396208

[B71] Sakai J. (2020). Core Concept: how synaptic pruning shapes neural wiring during development and, possibly, in disease. Proc. Natl. Acad. Sci. U.S.A. 117 (28), 16096–16099. 10.1073/pnas.2010281117 32581125 PMC7368197

[B72] Santos L. E. Beckman D. Ferreira S. T. (2016). Microglial dysfunction connects depression and Alzheimer's disease. Brain Behav. Immun. 55, 151–165. 10.1016/j.bbi.2015.11.011 26612494

[B73] Schafer D. P. Stevens B. (2015). Microglia function in central nervous System development and plasticity. Cold Spring Harb. Perspect. Biol. 7 (10), a020545. 10.1101/cshperspect.a020545 26187728 PMC4588063

[B74] Schober A. Böttner M. Strelau J. Kinscherf R. Bonaterra G. A. Barth M. (2001). Expression of growth differentiation factor-15/macrophage inhibitory cytokine-1 (GDF-15/MIC-1) in the perinatal, adult, and injured rat brain. J. Comp. Neurol. 439 (1), 32–45. 10.1002/cne.1333 11579380

[B75] Song A. Cheng R. Jiang J. Qu H. Wu Z. Qian F. (2024). Antidepressant-like effects of hyperoside on chronic stress-induced depressive-like behaviors in mice: gut microbiota and short-chain fatty acids. J. Affect. Disord. 354, 356–367. 10.1016/j.jad.2024.03.017 38492650

[B76] Strelau J. Sullivan A. Böttner M. Lingor P. Falkenstein E. Suter-Crazzolara C. (2000). Growth/differentiation factor-15/macrophage inhibitory cytokine-1 is a novel trophic factor for midbrain dopaminergic neurons *in vivo* . J. Neurosci. 20 (23), 8597–8603. 10.1523/JNEUROSCI.20-23-08597.2000 11102463 PMC6773071

[B77] Su F. Yi H. Xu L. Zhang Z. (2015). Fluoxetine and S-citalopram inhibit M1 activation and promote M2 activation of microglia *in vitro* . Neuroscience 294, 60–68. 10.1016/j.neuroscience.2015.02.028 25711936

[B78] Suswidiantoro V. Tang K. S. Rahman K. Ariestanti D. M. James R. J. Yan C. C. (2026). Natural compounds as multitarget agents in Alzheimer's diseases: evidence from *in vivo* and *in vitro* models. Front. Pharmacol. 17, 1766470. 10.3389/fphar.2026.1766470 41808871 PMC12968286

[B79] Takata S. Sakata-Haga H. Shimada H. Tsukada T. Sakai D. Shoji H. al. (2022). LIF-IGF axis contributes to the proliferation of neural progenitor cells in developing Rat cerebrum. Int. J. Mol. Sci. 23 (21), 13199. 10.3390/ijms232113199 36361987 PMC9659294

[B80] Troubat R. Barone P. Leman S. Desmidt T. Cressant A. Atanasova B. (2021). Neuroinflammation and depression: a review. Eur. J. Neurosci. 53 (1), 151–171. 10.1111/ejn.14720 32150310

[B81] Tsai V. W. W. Husaini Y. Sainsbury A. Brown D. A. Breit S. N. (2018). The MIC-1/GDF15-GFRAL pathway in energy homeostasis: implications for obesity, Cachexia, and other associated diseases. Cell Metab. 28 (3), 353–368. 10.1016/j.cmet.2018.07.018 30184485

[B82] Wainwright S. R. Galea L. A. (2013). The neural plasticity theory of depression: assessing the roles of adult neurogenesis and PSA-NCAM within the Hippocampus. Neural Plast. 013, 805497. 10.1155/2013/805497 23691371 PMC3649690

[B83] Wang Y. Zhen C. Wang R. Wang G. (2019). Growth-differentiation factor-15 predicts adverse cardiac events in patients with acute coronary syndrome: a meta-analysis. Am. J. Emerg. Med. 37, 1346–1352. 10.1016/j.ajem.2019.04.035 31029521

[B84] Wang Q. Wei H. C. Zhou S. J. Li Y. Zheng T. T. Zhou C. Z. (2022). Hyperoside: a review on its sources, biological activities, and molecular mechanisms. Phytother. Res. 36 (7), 2779–2802. 10.1002/ptr.7478 35561084

[B85] Worthen R. J. Garzon Zighelboim S. S. Torres Jaramillo C. S. Beurel E. (2020). Anti-inflammatory IL-10 administration rescues depression-associated learning and memory deficits in mice. J. Neuroinflammation 17 (1), 246. 10.1186/s12974-020-01922-1 32828124 PMC7443292

[B86] Yirmiya R. Goshen I. (2011). Immune modulation of learning, memory, neural plasticity and neurogenesis. Brain Behav. Immun. 25 (2), 181–213. 10.1016/j.bbi.2010.10.015 20970492

[B87] Yirmiya R. Rimmerman N. Reshef R. (2015). Depression as a microglial disease. Trends Neurosci. 38 (10), 637–658. 10.1016/j.tins.2015.08.001 26442697

[B88] Zhang T. Zhao L. Kuang R. (2025). Impact of hyperoside on the blood-brain barrier in rats with bacterial meningitis through the MicroRNA-155/Brain-Derived neurotrophic factor pathway. Neuroendocrinology 115 (9), 704–718. 10.1159/000547276 40618728

