# Supplementary material for: Effects of St. John’s Wort extract on the release of paracrine markers and neurite outgrowth in mouse hippocampal HT-22 neuronal cells co-cultured with SIM-A9 microglia
Source: Front Pharmacol. 2026 Oct 21;17:1916960. doi: 10.3389/fphar.2026.1916960 (PMC13731891; doi:10.3389/fphar.2026.1916960)
Supplement: Supplementary file 1 [file Supplementaryfile1.docx]

Supplementary Material

# 1 Supplementary data

-Enzyme-Linked Immunosorbent Assay (ELISA) raw results of

Brain-derived neurotrophic factors (BDNF), Leukemia inhibitory factor (LIF) also cholinergic differentiation factor (CDF), Growth/differentiation factor 15 (GDF-15), Interleukin 6 (IL-6), Interleukin 10 (IL-10) and Tumor necrosis factor alpha (TNF-α).

-Neurite Outgrowth/Viability

-Migratory Capacity using a scratch-wound closure assay

-Viability was determined using PrestoBlue™ Cell Viability Reagent

Open-Access-Repositorium-Link

# <https://open.uni-marburg.de/handle/openumr/11537>

# 2 Supplementary figures and tables

## 2.1 Supplementary Figure S4

## Mouse hippocampal HT-22 neuronal cells morphology and expression of N-Methyl-D-aspartic acid (NMDA)-Receptor

HT-22 cells show neurite outgrowth (A, white arrows) and expression of NMDA receptor (B, white arrows). Immunoreactivity (red) characterized using ICC (B) and counterstained using Hoechst 33258 (C). White bar: 50 µm.Zhao Z, Lu R, Zhang B, Shen J, Yang L, Xiao S, Liu J, Suo WZ (2012) Differentiation of HT-22 neuronal induces expression of NMDA receptor that mediates homocysteine cytotoxicity. Zhao et al., 2012. Neurological Research 34 (1):38-43

**Glutamate cytotoxicity**

The neuronal cell line HT-22 is sensitive to glutamate cytotoxicity via a non-receptor-mediated oxidative pathway. Viability assay mittels PrestoBlue^®^. Effect of glutamate toxicity in undifferentiated HT-22 cells. Viability was measured using PrestoBlue^®^ Cell Viability Reagent (Life Technologies). Control without treatement = 100% viability. Data are expressed in % as mean ± SEM

## 2.2 Supplementary Figure S5

**SIM-A9 cell morphology**

SIM-A9 displayed different morphologies in culture. Round cells without obvious processes (black arrows), bipolar (white arrows), or polygonal cells with multiple processes (yellow arrows), the distal ends of the processes often form lamellipodia (red arrows), suggesting that these cells are mobile. The morphological variations are commonly observed in cultured microglia depending on their activation states and culture conditions (Giulian and Baker, 1986. J Neurosci, 6: 2163–78.), SIM-A9 cells appeared to behave like their primary counterpart (Nagamoto-Combs et al., 2014. J. Neurosci. Methods, 233: 187-198).

**SIM-A9 phagocytic properties**

Similar to macrophages microglia cells have the ability to phagocyte biological debris after injury or infections in order to remove damaged cells or pathogens (Nagamoto-Combs et al., 2014. J Neurosci Methods, 233: 187-198). Phagocytosis of green fluorescent beads (white arrow) by mouse SIM-A9 cells without or after treatment (24h) with LPS E. Coli O111:B4 (1 µg/ml). (A) Microglia cells without LPS and (B) after LPS stimulation (24 h).

**Response to Lipopolysaccharide (LPS) stimulation**

Similar to macrophages SIM-A9 microglia cells after stimulation with LPS induced TNF-α release (Nagamoto-Combs et al., 2014. J Neurosci Methods, 233: 187-198).

**2.2**	**Supplementary Figure S6**

**Markers identified using Proteome Profiler™**

| **Name** | **Gene ID** | **Alternate name** | **References** |
| --- | --- | --- | --- |
| CXCL10 /IP-10 | 15945 | CRG-2, C7 | **Huang,** X., Yang, C., Huang, M. (2023). Protective mechanism of the EZH2/microRNA-15a-5p/CXCL10 axis in rats with depressive-like behaviors. *J. Chem. Neuroanat*. 132, 102283. doi: 10.1016/j.jchemneu.2023.102283. |
| GDF-15 | 23886 | MIC-1 | **Zang,** Y.; Zhu, Z., Xie, Y., Liu, Z., Yin, J., Yang, P., et al. (2022). Serum Growth Differentiation Factor 15 Levels Are Associated With Depression After Ischemic Stroke. J *Am. Heart Assoc.* 11(1), e022607. doi: 10.1161/JAHA.121.022607. |
| IGFBP-3 | 16009 |  | **Dai,** H., Goto, Y.I., Itoh, M. (2017). Insulin-Like Growth Factor Binding Protein-3 Deficiency Leads to Behavior Impairment with Monoaminergic and Synaptic Dysfunction. *Am. J. Pathol*. 187(2), 390-400. doi: 10.1016/j.ajpath.2016.10.011. |
| IL-10 | 16153 | CSIF | **Perez-Asensio,** F.J., Perpiñá, U., Planas, A.M., Pozas, E. (2013). Interleukin-10 regulates progenitor differentiation and modulates neurogenesis in adult brain. *J. Cell Sci*. 126 (Pt 18), 4208-19. doi: 10.1242/jcs.127803. |
| IL-1ra/IL-1F3 | 16181 | IL1RN | **DiSabato,** D.J., Yin, W., Biltz, R.G., Gallagher, N.R., Oliver, B., Nemeth, D.P., et al. (2022).. IL-1 Receptor-1 on Vglut2+ neurons in the hippocampus is critical for neuronal and behavioral sensitization after repeated social stress. *Brain Behav. Immun. Health*. 26, 100547. doi: 10.1016/j.bbih.2022.100547. |
| LIF | 16878 | CDF | **Xu,** L., Long, J., Shi, C., Zhang, N., Lv, Y., Feng, J., et al. (2018). Effect of leukocyte inhibitory factor on neuron differentiation from human induced pluripotent stem cell-derived neural precursor cells. *Int. J. Mol. Med.* 41(4), 2037-2049. doi: 10.3892/ijmm.2018.3418.  **Kurokawa,** K., Tsuji, M., Takahashi, K., Miyagawa, K., Mochida-Saito, A., Takeda, H., et al. (2020). Leukemia Inhibitory Factor Participates in the Formation of Stress Adaptation via Hippocampal Myelination in Mice. *Neuroscience.* 446, 1-13. doi: 10.1016/j.neuroscience.2020.08.030. |
| MMP-2 | 17390 | Gelatinase A | **Lively,** S., Schlichter, L.C. (2013). The microglial activation state regulates migration and roles of matrix-dissolving enzymes for invasion. J Neuroinflammation. 10, 75. doi: 10.1186/1742-2094-10-75. |
| Pentraxin 3/TSG-14 | 19288 | PTX3 | **Zhou,** C., Chen, H., Zheng, J.F., Guo, Z.D., Huang, Z.J., Wu, Y., et al. (2020). Pentraxin 3 contributes to neurogenesis after traumatic brain injury in mice. *Neural Regen. Res.* 15(12), 2318-2326. doi: 10.4103/1673-5374.285001. |
| VCAM-1/CD106 | 22329 |  | **Lehtinen,** M.K. (2012). Adult neurogenesis: VCAM stems the tide. *Cell Stem Cell.* 11(2):137-8. doi: 10.1016/j.stem.2012.07.012. |

CXCL10: C-X-C Motif Chemokine Ligand 10

GDF-15: Growth Differentiation Factor 15

IGFBP-3: Insulin Like Growth Factor Binding Protein 3

IL-10: Interleukin 10

IL-1ra: Interleukin-1 receptor antagonist

LIF: Leukemia inhibitory factor

MMP-2: Matrix metalloproteinase-2

Pentraxin 3: Pentraxin-related protein PTX3

VCAM-1: Vascular cell adhesion protein 1
